# Effect of Storage Conditions on the Stability of Polyphenols of Apple and Strawberry Purees Produced at Industrial Scale by Different Processing Techniques

**DOI:** 10.1021/acs.jafc.2c07828

**Published:** 2023-01-27

**Authors:** Gabriela L. Salazar-Orbea, Rocío García-Villalba, María J. Bernal, Alberto Hernández-Jiménez, Jose A. Egea, Francisco A. Tomás-Barberán, Luis M. Sánchez-Siles

**Affiliations:** † Quality, Safety, and Bioactivity of Plant-Derived Foods, Centro de Edafología y Biología Aplicada del Segura-Consejo Superior de Investigaciones Científicas (CEBAS-CSIC), 30100 Murcia, Spain; ‡ Research and Nutrition Department, Hero Group, 30820 Alcantarilla, Spain; § Institute for Research and Nutrition, Hero Group, 5600 Lenzburg, Switzerland; ∥ AMC Natural Drinks Group, 30100 Murcia, Spain

**Keywords:** food-processing, reprocessing, storage, strawberry puree, apple puree, polyphenols

## Abstract

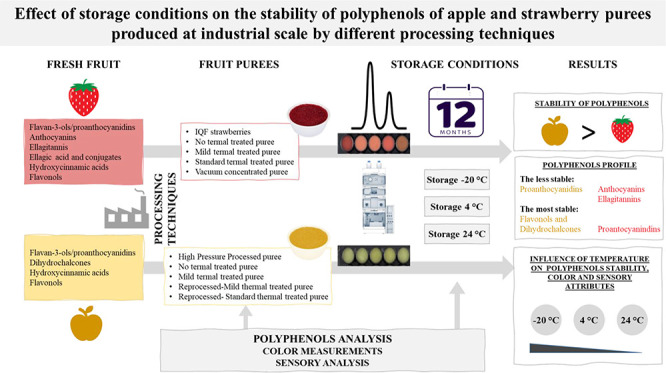

During a food product’s life, storage conditions affect its composition of nutrients, bioactive compounds, and sensory attributes. In this research, strawberry and apple purees were selected as a model to examine how the storage of various purees industrially produced with different technologies affect the bioactive phenolic compounds, color, and sensory attributes. Specifically, fruit products processed on an industrial scale by different technologies including freezing, thermal treatment (mild and standard), and high-pressure processing were studied, as well as storage for up to 12 months at −20, 4, and 24 °C. In strawberry puree, storage conditions had a stronger impact on phenolic compound levels, particularly on anthocyanins, whereas in apple puree, the initial processing techniques exerted a greater influence than storage conditions, mainly caused by the hot or cold crushing processes. In general, proanthocyanidins were the major phenolic group and the most stable during storage, while anthocyanins were the group most affected by both processing and storage. Apple flavonols and dihydrochalcones were quite stable, while strawberry ellagitannins suffered higher degradations during storage. Through our analysis, it is found that during storage, the stability of polyphenols in each fruit is different, and processing and storage can be either detrimental or even beneficial. The selection of the ideal storage conditions (time and temperature) is a key factor to maintaining the polyphenol content in sensitive fruits such as strawberries. However, storage conditions are in some cases more important to minimizing the polyphenol losses than how the product is processed.

## Introduction

Consumers generally believe that the industrial processing of foods makes these not only less natural but also less healthy.^1−3^ However, processing makes food more edible, palatable, and safe, extending at the same time the shelf life of products,^4^ which in turn, is fundamental to reducing food waste.^5,6^ But not all types of processing are the same; it is necessary to differentiate the degree of processing of the products. A recent publication in the field of metabolomics has led to the determination of some markers that can help identify the degree of processing in strawberry and apple products.^7^

Shelf life refers to the length of time that a food product can be stored without becoming unsuitable for human consumption in terms of its safety, nutritional attributes, and sensory characteristics.^8^ It can range from a few days to several years, depending on food formulation, degree and type of processing, storage conditions (time and temperature of storage), and packaging type.^9,10^ Processing and storage conditions play a major (positive or negative) role in determining shelf life, particularly in fruit derived products.^10−12^ For example, after 35 days of storage at 6 °C, almost no vitamin C was left in any of the strawberry purees processed with different high-pressure processing conditions.^13^ On the contrary, recent findings from Salazar-Orbea et al.^14^ evidence that just after processing, the phenolic compounds and the sensory characteristics of fresh strawberry and apple purees were minimally affected when mild or standard treatments were applied on an industrial scale.

Interestingly, even though polyphenols in fruits are associated with important health benefits,^15^ they are rarely used as a marker to determine the shelf life of fruits, most likely because of the complexity of conducting the required analyses.^16^ The rate of degradation of these components (phenolics) in fruit based products can be significantly affected by the food matrix (e.g., jam, juice, or puree), fruit type, fruit variety, maturity stage, processing degree (type and conditions), storage conditions, and packaging material.^10,17,18^

Previous studies examining how processing and storage influence phenolic compounds and sensory properties of fruit based products have mostly relied on laboratory scale experiments which simulate the industrial context.^19−23^ However, the conditions (e.g., temperature and times) as well as the equipment used in real (industrial) settings significantly differ from those used in laboratory settings, thus limiting the applicability and implementation of the results in a real (industrial) context. For example, several laboratory scale studies have ignored the deaeration step of puree,^22,24−26^ which represents an essential processing stage that maximizes the oxidative stability of phenolic compounds during storage.

The aim of the current research was to examine the extent to which the storage conditions (temperature and time) influence the levels of bioactive phenolic compounds, color, and sensory attributes of strawberry and apple purees produced under real food production systems by different processing techniques. Importantly, both fruits represent a good source of bioactive compounds such as polyphenols.^27,28^

## Materials and Methods

### Chemicals and Samples

Standards of (+)-catechin, (−)-epicatechin, *p*-coumaric acid, ferulic acid, ellagic acid, quercetin 3-rutinoside, quercetin, cyanidin 3-glucoside, phloridzin, and phloroglucinol with purity >99% were purchased from Sigma-Aldrich (St. Louis, MO, USA). Castalagin was kindly provided by Dr. S. Quideau (Bordeaux, France). Methanol and acetonitrile were from J.T. Baker (Deventer, The Netherlands), formic acid from Honeywell (Barcelona, Spain), and hydrochloric acid and sodium acetate from Panreac (Barcelona, Spain). Ascorbic acid was from Acros Organic (Geel, Belgium). Water was deionized using a Milli-Q-system (Millipore, Bedford, MA, USA).

Strawberries (*Fragaria x ananassa*) cv. Primoris were from Huelva (Spain) and harvested ripe (80% red) in June 2018. Apples (*Malus domestica*) cv. Golden Delicious were from Zaragoza (Spain) and harvested in November 2019.

### Processing and Storage of Strawberry and Apple Products

**Figure 1:**
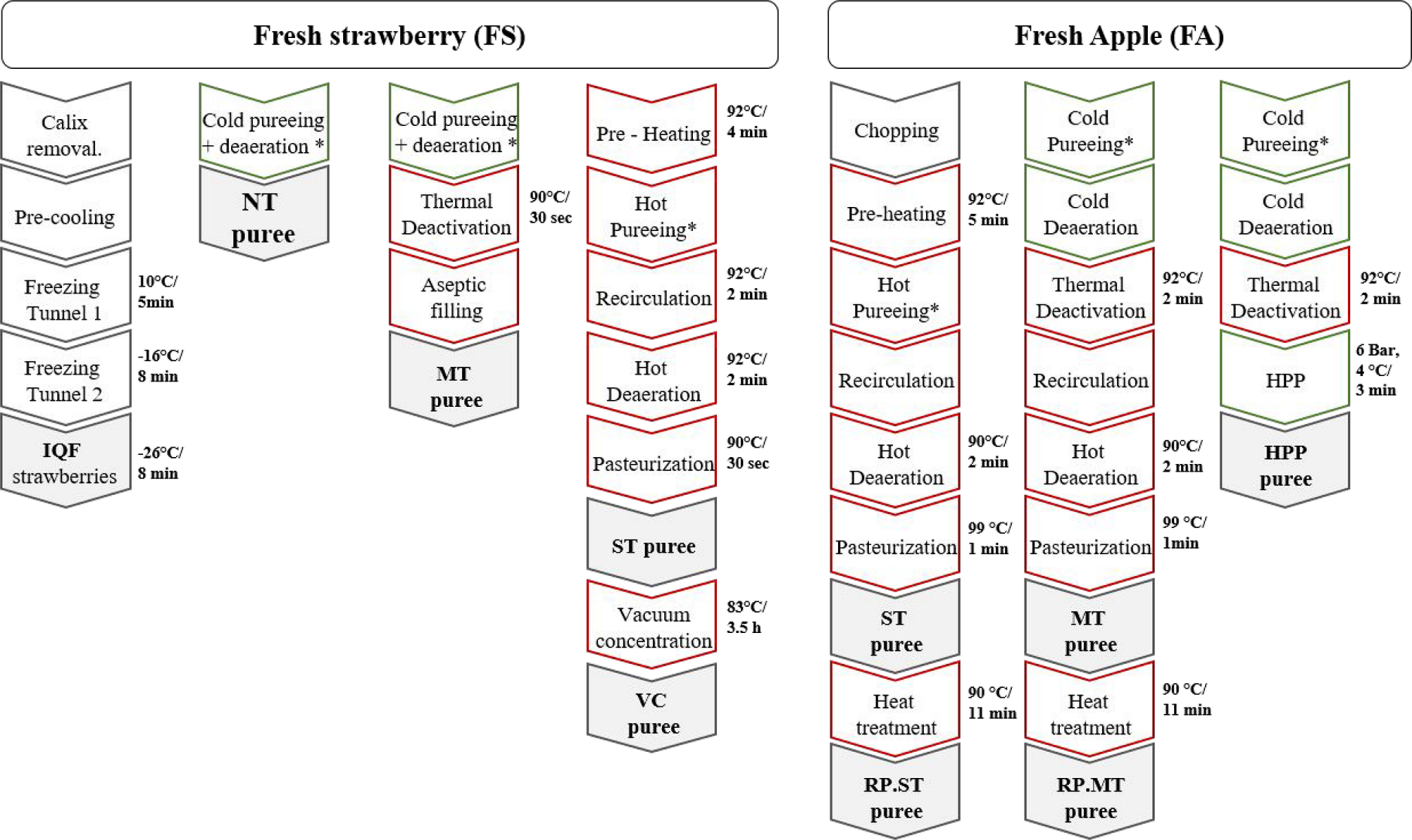
Scheme of the different processing technologies’ conditions applied to strawberries and apples. IQF, individual quick freezing; NT, nonthermally treated; MT, mild thermal treatment; ST, standard thermal treatment; VC, vacuum concentrated; RP, reprocessed; HPP, high pressure processed; *Removal of peel and seeds.

Strawberries and apples were industrially processed with different technologies and conditions (Figure 1) as previously described by Salazar-Orbea et al.^14^ and stored for 12 months at −20, 4, and 24 °C according to the scheme shown in Table 1. This experiment includes two studies, which analyze the storage as follows: Study 1, individually frozen strawberries (IQF) and strawberry purees produced by cold pureeing without heat treatment (NT); cold pureeing + mild thermal treatment (MT); hot pureeing + standard thermal treatment (ST); and standard thermal treatment + vacuum concentration (VC). Study 2, apple purees obtained by cold pureeing with enzyme inactivation + high pressure processing (HPP); cold pureeing + mild thermal treatment (MT); and hot pureeing + standard thermal treatment (ST). In addition, the storage of reprocessed (RP) apple purees [MT and ST samples stored at 24 °C for six months and subjected to an additional reprocessing thermal treatment (90 °C/11 min)] RP.MT and RP.ST was evaluated to simulate the use of stored apple purees as raw materials to elaborate upon other products.

**Table 1:** Storage Conditions Applied to Strawberry and Apple Purees Obtained after Different Industrial Processing Technologies^a^

		storage temperature		
fruit	sample	–20 °C	4 °C	24 °C
strawberry	IQF	●		
	NT puree	●		
	MT puree	●	●	●
	ST puree	●	●	●
	VC puree			●
apple	HPP puree		●	
	MT puree	●	●	●
	ST puree	●	●	●
	RP.MT puree			●
	RP.ST puree			●

a IQF, individual quick freezing; NT, nonthermal treated; MT, mild thermal treated; ST, standard thermal treated; VC, vacuum concentrated; HPP, high pressure processed; RP, reprocessed.

Processed strawberry and apple purees were aseptically filled into low permeability (<0.02 cc/m^2^/day) double membrane aseptic bags [outer membrane, PE/Met PET/PE of 102 μm; inner membrane, CoEx PE/EVOH/PA/PE of 90 μm (ARAN Packaging, Spain)] and stored for 12 months at −20, 4, and 24 °C (Table 1). IQF strawberries and NT strawberry puree were stored only at −20 °C, while VC strawberry puree was filled into vacuum-sealed glass jars and kept at 24 °C. HPP apple puree was preserved only at 4 °C. In the case of the apple reprocessed purees, these were vacuum filled in glass jars and stored at 24 °C, which is a common market standard. All stored samples were analyzed at 2, 6, and 12 months. Three replicates of each processing technique and storage condition were analyzed. To remove the moisture, all of the samples were lyophilized and ground into powder using a dry bean blender to homogenize the sample before extraction.

### Analysis of Phenolic Compounds

Different families of phenolic compounds were identified and quantified by HPLC-DAD-ESI-MS using the methods previously described in Salazar-Orbea et al.^14^ Phenolic compounds were extracted from strawberry samples (50 mg) with 1 mL of methanol/water/acetic acid (70:29:1, v/v/v) and from apple samples (50 mg) with 1 mL of ethanol/water (70:30, v/v). The samples were homogenized in a vortex for 1 min and then sonicated for 30 min at room temperature. Subsequently, samples were centrifuged for 15 min at 20 627*g* at 12 °C (Thermo Scientific Sorvall ST 16, Germany). The resultant supernatant was filtered through a 0.22 μm filter. Compounds were identified by their UV spectra, retention time, and MS spectra and quantified using UV detection chromatograms recorded at 280 nm (ellagitannins, flavan 3-ols, and proanthocyanidins), 320 nm (hydroxycinnamates), 360 nm (flavonols and ellagic acid conjugates), and 520 nm (anthocyanins). For the analysis of proanthocyanidins, a phloroglucinolysis method according to Kennedy and Jones was used to quantify their constitutive units and to determine the mean degree of polymerization (mDP).^29,30^ 800 μL of a solution of 0.1 N HCl in MeOH containing 5 g/L phloroglucinol and 10 g/L ascorbic acid was added to 0.8 g of lyophilized powdered sample. The mixture was incubated at 50 °C for 20 min with constant steering. Subsequently, 1 mL of 40 mM sodium acetate was added to stop the reaction. Finally, the samples were centrifuged for 10 min at 20 627*g* (Thermo Scientific Sorvall ST 16, Germany), and the supernatant was filtered through a 0.22 μm PVDF filter. All extractions were performed and analyzed in triplicate.

### Color Measurements

The measurement was made on the CIEL*a*b* system, using a CR-400 Chromameter (Konica Minolta, Japan) under the following conditions: illuminant C, observer 2°, and 8 mm of illumination area. Color was reported as the total color difference (Δ*E*) coefficient and was calculated with the following equation Δ*E* = ((*L*_0_^*^ – L*)^2^ + (*a*_0_^*^ – a*)^2^ + (*b*_0_^*^ – b*)^2^))^1/2^, where *L*_0_^*^, *a*_0_^*^, and *b*_0_^*^ were the values for the sample after processing.

### Sensory Analysis

The sensory analysis of the purees was determined using a nine-point hedonic scale derived from Sukanya et al.^31^ Scores ranged from 1 = “I dislike it extremely” to 9 = “I like it extremely”. The attributes evaluated were color, viscosity, aroma, flavor, and overall evaluation. In general, an overall evaluation higher than 5 was considered an adequate indicator of acceptability.^32^ The evaluations were made by a semitrained sensory panel of 10 persons (age 25–60 years). Samples were sensory-analyzed after processing and after 2, 6, and 12 months of storage at −20, 4, and 24 °C. First, the samples (about 50 mL) were allowed to reach room temperature and presented to the panel randomly in transparent plastic cups. Then the panelists tested the attributes previously mentioned and wrote their answer according to the codes presented. Water was offered for cleaning the aftertaste between samples.

### Statistical Analysis

A principal component analysis (PCA) was performed independently on strawberries and apples to study the clustering patterns of the samples. The data matrix consisted of the samples processed by the different techniques, just after processing (0) and after storage at −20, 4, and 24 °C for 2, 6, and 12 months, and the total amount of the different polyphenol families as variables. Data were scaled and centered prior to PCA. This standardization to the same scale avoids some variables becoming dominant just because of their large amount. PCA biplot graphs were constructed for the visual interpretation of the results. A one-way analysis of variance (ANOVA), followed by comparisons using a Tukey test with a confidence level of 0.05, was performed to evaluate the effect of storage time at each subjected temperature on the phenolic content, color parameters, and sensory attributes of the different treated samples. All data analyses were conducted using R software, version 4.0.2.^33^

## Results

### Quantification of Polyphenols in Strawberry and Apple Samples

Phenolic compounds from different families (Table S1), previously determined in strawberry and apple samples subjected to different processing technologies,^14^ were quantified during storage at different times and temperatures (Tables S2, S3, and S4). Individual compounds quantified in each family are shown in Table S1. More information about these compounds was previously reported (Salazar-Orbea et al.). In strawberries, 11 phenolic compounds were quantified: three ellagitannins, two hydroxycinnamic acids, one flavonol, two ellagic acid conjugates, and three anthocyanins. In apples, 12 phenolic compounds were quantified including two dihydrochalcones, four hydroxycinnamic acids, and six flavonols. Proanthocyanidins were determined after the phloroglucinolysis reaction, quantifying the flavan-3-ol monomers of catechin, epicatechin, and afzelechin in strawberries and catechin and epicatechin in apple samples with methodologies previously described.^29,30^

### Exploratory Analysis of Data

As a first exploratory step, an unsupervised principal component analysis (PCA) was applied to visualize similarities or differences among samples based on their phenolic content and to identify data clustering trends. Importantly, this preliminary evaluation of data was developed considering all the strawberry and apple samples subjected to different processing conditions on an industrial scale and stored at different times and temperatures compatible with the real food production system. Barltlett’s test of sphericity was significant for strawberry (2.41e-5) and apple (2.16e-28) samples, which verified the overall significance of all correlations within the correlation matrix. The Kaiser–Meyer–Olkin (KMO) value was 0.67 for strawberry and 0.51 for apple samples, which indicated the relationships among variables.

**Figure 2:**
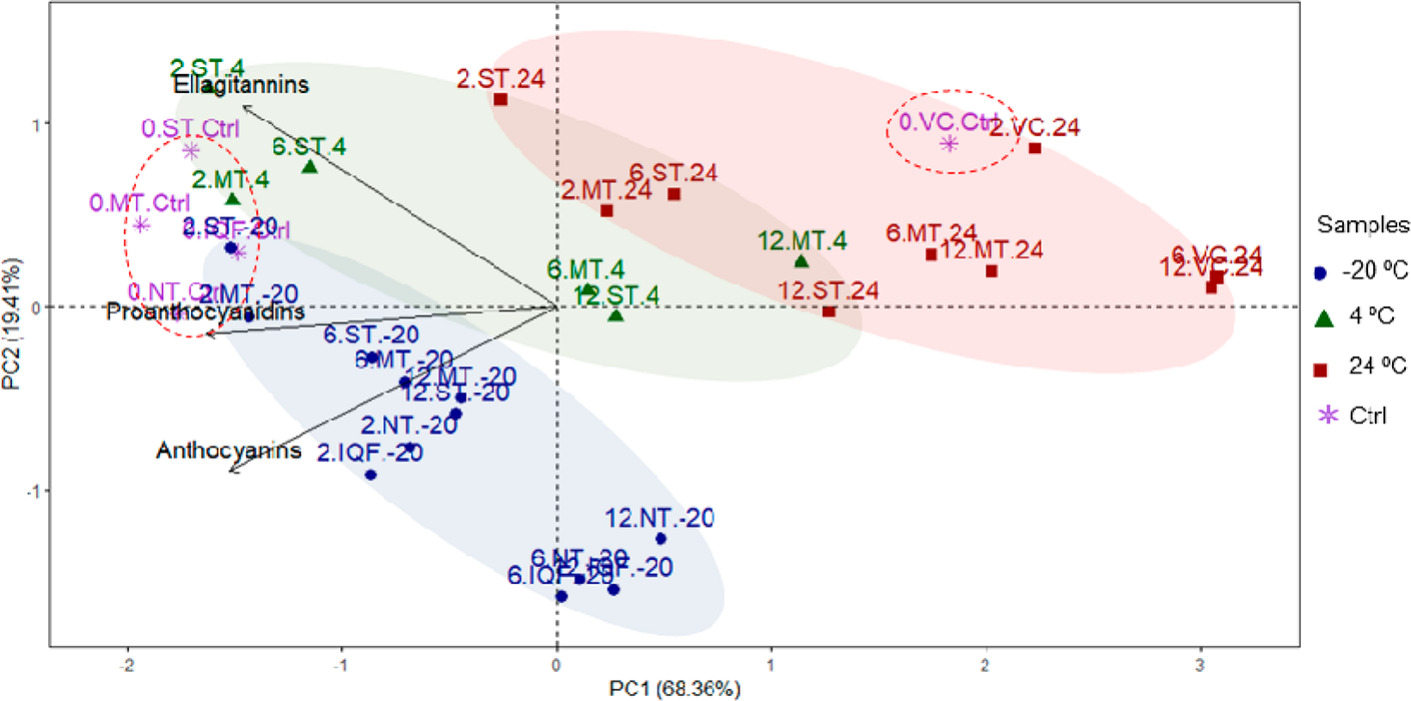
Principal component analysis (PCA) of strawberry samples (before processing, 0.Ctrl; after processing by IQF, individual quick freezing; NT, no heat treatment; MT, mild treatment; ST, standard treatment; VC, vacuum concentration) after 2, 6, and 12 months of storage at −20 (blue), 4 (green), and 24 °C (red). Dashed lines show unprocessed control samples.

In strawberries, the PCA model was built considering the amount of the most abundant phenolic compounds, namely, proanthocyanidins, ellagitannins, and anthocyanins, and with scaled data to ponder the same weight to all the variables. Other minor compounds such as hydroxycinnamic acids, flavonols, and ellagic acid conjugates, that represented around 3.4%, 2.7%, and 0.8% of the total phenolic content, respectively, were not included in the scaled PCA due to their low influence on the total amount. Two principal components (PC1 and PC2) explained 87.8% of total variance and together were capable of separating the samples into three groups according to the storage temperature (Figure 2). A clear separation between the clusters stored at 24 and −20 °C was observed, while a cluster stored at 4 °C showed a high degree of overlapping with the other two groups. Samples obtained just after the different processing (0.IQF.Ctrl, 0.NT.Ctrl, 0.MT.Ctrl, 0.ST.Ctrl) were grouped together (red circle in Figure 2) showing similar phenolic profiles and the relatively low impact of these treatments. Only the samples subjected to the more extreme conditions (0.VC.Ctrl) appeared notably separated. The effect of storage temperature was visible in PC1:24 °C (positive values, on the right) and 4 and −20 °C (negative values, on the left). Samples (VC, MT, and ST) stored at 24 °C showed lower concentrations of all phenolic compounds and especially VC puree, which was located further right on PC1, even in the samples obtained immediately after processing (0.VC.Ctrl).

VC samples showed the highest PC1 values followed by MT and ST, and each one at 6 and 12 months of storage was located at higher PC1 values compared to storage after 2 months. These results confirmed the expected changes as the higher the temperature and the longer the processing time, the more phenolic compounds were degraded. All treatments stored at −20 °C were very close to the control cluster, indicating that the phenolic compounds were well preserved at this temperature. Within this cluster, NT and IQF samples (specially at 6 and 12 months) were grouped together at the bottom (lower PC2 values), evidencing low levels of ellagitannins and a better retention of anthocyanins. Samples stored at 4 °C showed different behaviors depending on the treatment (ST or MT) and storage time.

**Figure 3:**
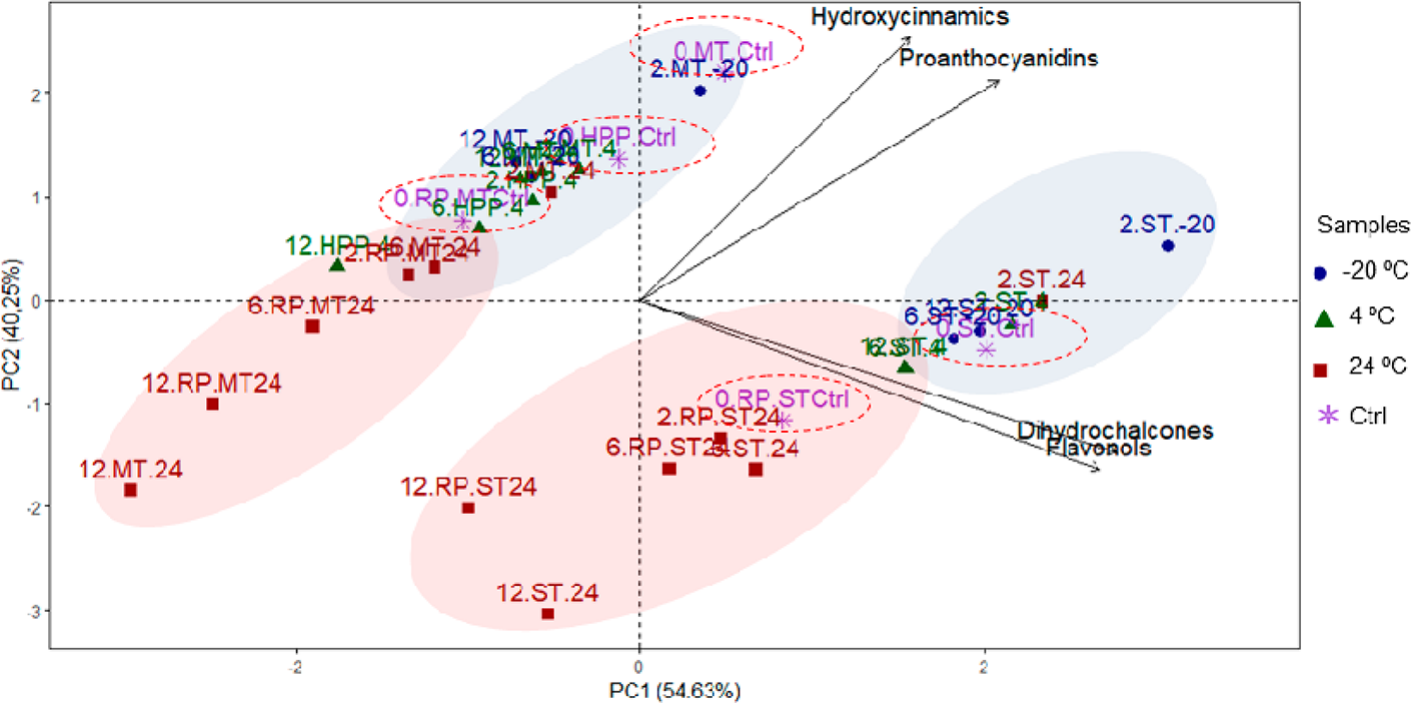
Principal component analysis (PCA) of apple samples (before processing, 0.Ctrl; after processing by MT, mild treatment; HPP, cold extraction + HPP; ST, standard treatment; RP, reprocessed) after 2, 6, and 12 months of storage at −20 (blue), 4 (green), and 24 °C (red). Dashed lines show unprocessed control samples.

In apples, two principal components PC1 and PC2 explained the 94.8% variation in all the samples (Figure 3). Samples were clustered into two groups according to the technological treatment applied. The first one comprising ST samples was located on the right-bottom side of the plot, whereas the second cluster grouped MT and HPP samples and was located on the left-top side of the plot. PC1 (which explained 54.6% of total variance) was mainly associated with the industrial treatments, as in MT and HPP samples the fruits were cold crushed, while ST processed the fruits under hot crushing. The cluster with ST samples mainly located at PC1 > 0 was characterized by a higher content of flavonols and dihydrochalcones compared to MT and HPP. In each cluster, samples were grouped along PC2 (40.2% of total variance) according to the storage temperature. Samples stored at −20 and 4 °C clustered at higher PC2 values, compared to those stored at 24 °C and were associated with higher concentrations of proanthocyanidins and hydroxycinnamic acids. The effect of processing was observed mainly in PC1, whereas time and storage conditions influenced PC2.

### Influence of Storage on the Phenolic Composition

**Figure 4:**
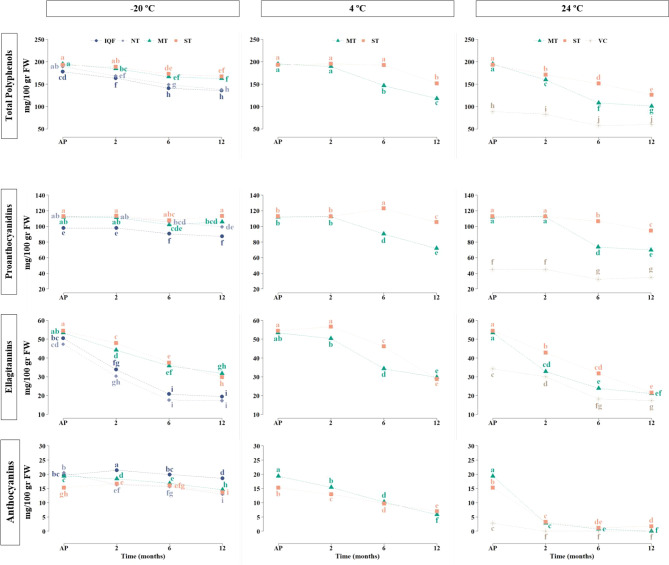
Strawberry purees. Mean values of total polyphenols and the phenolic groups proanthocyanidins, ellagitannins, and anthocyanins. Strawberries industrially processed by IQF, individual quick freezing; NT, no heat treatment; MT, mild thermal treatment; ST, standard treatment; VC, vacuum concentration. Samples collected just after processing (AP), and after 2, 6, and 12 months of storage at −20, 4, and 24 °C. Different letters within the same phenolic group and storage temperature are significantly different at *p* < 0.05.

**Figure 5:**
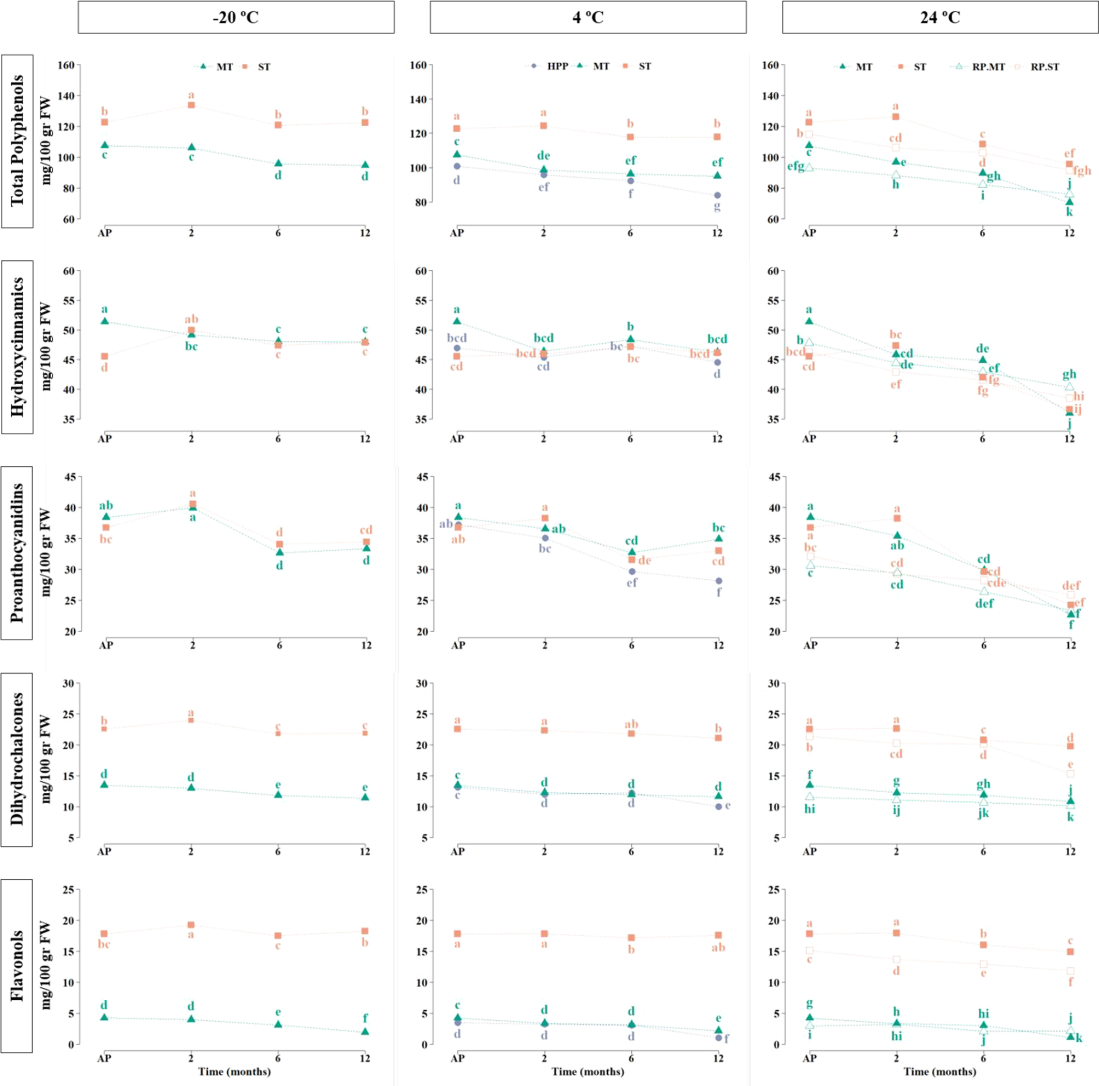
Apple purees. Mean values of total polyphenols and the phenolic groups, hydroxycinnamic acids, proanthocyanidins, dihydrochalcones, and flavonols. Apples industrially processed by MT, mild treatment; ST, standard treatment; HPP, high pressure processing; RP.MT, mild treatment + reprocessing; RP.ST, standard treatment + reprocessing. Samples collected just after processing (AP) and after 2, 6, and 12 months of storage at −20 °C, 4 °C, and 24 °C. Different letters within the same phenolic group and storage temperature are significantly different at *p* < 0.05.

A more in-depth analysis was conducted to study the influence of industrial processing and storage conditions (temperature and time) on the phenolic composition of strawberry and apple samples produced by different technological treatments on an industrial scale (Figures 4 and 5). The change in the polyphenol concentration of the samples stored at different times and temperatures was calculated as a percentage with respect to the samples just after processing and is shown in Tables S2, S3, and S4.

#### Strawberries and Strawberry Puree Samples

##### Proanthocyanidins

After storage at −20 °C, there were no significant differences in proanthocyanidin concentration in MT and ST samples (Figure 4), whereas IQF and NT samples experienced slight reductions from six months, reaching a decrease of around 11% at 12 months. MT and ST samples were also stable after 2 months of storage at 4 and 24 °C, but after 6 months a reduction was observed that continued after 12 months of storage. Although MT at 4 °C preserved proanthocyanidins better than at 24 °C until the sixth month (19.2% and 34.2% of reduction, respectively), at the end of storage, MT exhibited similar losses of about 36% at both storage temperatures.

Interestingly, the stability of proanthocyanidins was higher in samples processed by ST compared to MT under all the conditions (final loss 16.4% in ST and 37.6% in MT at 24 °C). The initial concentration of proanthocyanidins in VC was already significantly low (45.09 mg/100 g FW) compared with the other treatments, and it was further reduced during storage at 24 °C, exhibiting final losses of 22.5%. In general, a decrease in the mDP (mean degree of polymerization) was observed in the course of storage, except for IQF and NT purees, where it remained stable.

##### Ellagitannins

Ellagitannin levels in strawberry products (Figure 4) were similar after different processing techniques, except in VC samples that showed lower concentrations. Significant losses of ellagitannins were observed during storage (Table S2). In samples stored at −20 °C, ellagitannin degradation was observed from 2 months, being more pronounced for IQF and NT (losses of 61.5% and 63.5%, respectively, at the end of storage with concentrations around 18 mg/100g FW) compared to MT and ST (final losses of 40.4% and 45.3% and concentrations around 29 mg/100g FW). Ellagitannins were better preserved in MT and ST kept at 4 °C than in those stored at −20 °C up to the sixth month. However, at the end of storage, MT and ST recorded similar losses at both temperatures reaching similar concentrations around 29 mg/100 g FW. Overall, MT experienced a faster degradation of ellagitannins during storage compared to ST, although the final concentration at the end of storage was the same (21 mg/100g FW). More severe degradations of MT and ST samples were observed at 24 °C in all storage times, and again MT showed a faster degradation, although at the end of the storage similar losses of around 60% were observed for both treatments. In VC samples, there was an important initial degradation, exclusively attributed to treatment, but the final losses were lower (around 49%). These samples exhibited the lowest ellagitannin concentrations after storage (17.5 mg/100 g) similar to those obtained at −20 °C with NT and IQF.

##### Anthocyanins

These were the most susceptible phenolic compounds to processing and storage temperatures (Figure 4). As could be expected, anthocyanins were better preserved at −20 °C. During storage at this temperature, IQF showed the lowest losses in anthocyanins (5.5%), followed by ST, MT, and NT, which recorded losses of 10.4%, 24.7%, and 36.5%, respectively, at the end of storage (Table S2). The processing treatments with higher temperatures showed lower losses of anthocyanins during storage, but as they started from smaller amounts the final concentration was similar for all of them, approximately 14 mg/100 g FW. A higher degradation of anthocyanins after 2 months was observed in MT and ST samples stored at 4 °C, resulting in losses of 54.3% for ST and 69.7% for MT at the end of storage and concentrations of 5.87 and 6.99 mg/100 g FW, respectively. The most severe anthocyanin losses were evidenced in MT and ST purees stored at 24 °C, where mean losses over 80% occurred after the second month of storage. As was reported for the other phenolic groups, VC produced the highest impact on anthocyanin levels after processing and during storage; in this case total losses were recorded from the second month of storage.

##### Minor Components (Ellagic Acid and Conjugates, Hydroxycinnamic Acids, and Flavonols)

The concentrations of these compounds were around 1 and 7 mg/100 g FW, and therefore their contribution to the total amount of polyphenols was very low (Table S3). The amount of ellagic acid increased at all storage temperatures and was especially relevant after 2 months of storage. Interestingly, the highest increase was observed with NT samples after storage at −20 °C, similar to those obtained for MT and ST samples stored at 24 °C. As for the hydroxycinnamic acids, they were quite stable at all temperatures with a slight decrease after 12 months of storage. Only NT samples at −20 °C and VC samples at 24 °C suffered a gradual decrease during storage. In the case of flavonol content, the samples stored at −20 °C were quite stable, except for the high increase observed for IQF samples stored for 12 months. MT samples at 4 and 24 °C suffered a slight decrease after 2 months, but after that, they remained stable. Conversely, an increase in flavonols was observed in ST samples stored at 4 and 24 °C for 2 and 6 months, which then decreased until the end of the storage. Also, an increase was observed in VC samples after 2 months that kept stable until the end of the storage.

##### Total Polyphenols

At the end of the storage (Figure 4), the average losses of TP in MT and ST stored at −20 °C were minimal (∼15%), whereas NT and IQF showed a higher degradation of around 25%. MT samples stored at 4 °C were stable until the second month of storage, but losses of 24.7% and 39.5% were observed after six and 12 months of storage, respectively. ST samples were more stable, showing no changes until six months of storage and total losses of 21.4% at the end of storage. An earlier (from two months) and more intense degradation was detected in MT and ST samples stored at 24 °C, and even more in MT than in ST. Among the samples stored at 24 °C, VC exhibited the lowest levels of TP.

#### Apple Puree Samples

##### Hydroxycinnamic Acids

No important changes were observed in samples stored at −20 and 4 °C (Figure 5). MT puree showed losses of about 4.3% and 9.7% after the second month of storage, and then the concentration remained stable until the end. Interestingly, hydroxycinnamic acid concentration increased about 9.7% in ST puree kept at −20 °C after the second month of storage. Nonetheless, no significant differences in hydroxycinnamic acids were reported in ST and HPP purees stored at 4 °C. In samples stored at 24 °C, MT caused losses of 10.7%, 12.7%, and 29.9% at 2, 6, and 12 months, respectively, whereas ST preserved hydroxycinnamic acids until the second month of storage, to after decrease 7.7% and 19.6% at 6 and 12 months, respectively. At the end of storage, no significant differences in hydroxycinnamic acid concentration (around 36 mg/100 g) were observed between MT and ST treatments because MT started from higher initial concentrations (Table S4). RP.MT and RP.ST showed the same degradation degree on hydroxycinnamic acids ending with mean losses of 16% and concentrations around 40 mg/100 g FW. Remarkably, RP.MT had a significantly higher hydroxycinnamic acid concentration (40.32 mg/100 g FW) than MT (35.99 mg/100 g FW) at the end of storage. There were no significant differences in hydroxycinnamic acids between ST and RP.ST purees at the end of storage.

##### Proanthocyanidins

Similar to the hydroxycinnamic acids, proanthocyanidin concentration slightly increased in ST purees under all of the storage conditions at the second month, although this increase was only significant in the samples stored at −20 °C (Figure 5). Overall, no significant differences were observed in proanthocyanidin levels up to the second month of storage in MT and ST kept at all temperatures (Table S4) and HPP at 4 °C. MT stored at −20 and 4 °C and ST stored at 4 °C exhibited similar losses of around 14% after 6 months and 10% after 12 months, reaching similar concentrations at the end of storage (≈ 34 mg/100 g FW). The reduction in ST samples stored at −20 °C was lower, but the final concentration remained similar. Among the treatments stored at 4 °C, HPP showed the highest degradation of about 24% at the end of storage (28.16 mg/100 g FW).

Samples stored at 24 °C produced the greatest degradation on proanthocyanidins starting from month 6 with losses of 40.9% and 33.9% for MT and ST, respectively, at the end of storage. RP.MT and RP.ST samples showed less degradation (23.6% and 19.4%, respectively, at the end of storage), but as they started with lower initial concentrations, no significant differences on proanthocyanidins were observed at the end of storage between all the treatments at 24 °C (MT, ST, RP.MT, and RP.ST; final concentration between 23 and 26 mg/100 g FW). In the case of the mDP a slight reduction was observed over time in all of the treatments regardless of the storage temperature.

##### Dihydrochalcones

Dihydrochalcones were quite stable during storage under all conditions (Figure 5). Only slight reductions were observed after 6 and 12 months of storage, always higher in MT than in ST. The greatest degradation was observed in HPP puree stored at 4 °C, at about 23.7% at the end of storage. Among the purees stored at 24 °C, MT and ST exhibited losses of 19.5% and 12.3%. In the reprocessed samples, a lower degradation was observed in RP.MT (12.4%) compared to MT (19.5%), but similar concentrations were obtained at the end of storage (around 10 mg/100 g FW). On the contrary, RP.ST showed a higher degradation and lower final concentration (15.3 mg/100g FW) than ST (19.78 mg/100g FW).

##### Flavonols

Results in this regard were similar to those obtained with the dihydrochalcones. A slight but significant increase was observed in ST puree kept at −20 °C at the second month of storage (Figure 5). However, when the sample was kept at 4 °C, no significant changes in flavonols were registered over time. Regarding MT and HPP, given that they had low initial concentrations of flavonols, even minimal changes in the net concentration seemed to be substantial percentage losses while being stored. The concentration of flavonols in MT puree after processing was 4.01 mg/100 g FW, which at the end of storage at −20 and 4 °C experienced losses of about 50%. Higher losses (69.2%) were observed in HPP samples stored at 4 °C, having a final concentration of 1.08 mg/100 g FW. In the samples stored at 24 °C, ST retained the flavonols relatively well, losing only 16.1% compared with the puree after processing. Conversely, MT kept at 24 °C led to reductions of 73.6% at the end of storage. As in dihydrochalcones, RP.ST led to higher losses of flavonols than ST (21.7%), and RP.MT showed a lower degradation (27.3%) compared with MT (73.6%).

##### Total Polyphenols

Significant increases in total polyphenols were evidenced after 2 months on ST stored at −20 and 4 °C, and then a slight decrease in both temperatures was observed after 6 and 12 months. MT exhibited average losses of 11% after months 6 and 12 at −20 and 4 °C (Figure 5). Within the samples stored at 4 °C, HPP led to slightly higher losses of about 16.8% on total polyphenols at the end of storage. Samples stored at 24 °C experienced the highest degradation among all the storage conditions, especially after 6 months. At the end of storage, ST showed losses of 22.0%, while MT induced 34.3% TP losses. In general, ST preserved better polyphenols under all storage conditions. In both reprocessed purees, lower losses of approximately 19% were observed, although the final concentration was similar because the initial concentration was also lower.

#### Color Changes during Storage

The color differences (Δ*E*) of strawberry and apple samples after processing and during storage (2, 6, and 12 months) are shown in Tables S5 and S6.

Overall, in strawberry purees, Δ*E* values increased as storage time progressed. Changes in Δ*E* were less noticeable in samples stored at −20 °C. Among the samples stored at 4 °C, MT was the treatment with the greatest Δ*E* at the end of storage. MT and ST samples stored at 24 °C resulted in higher rates of color difference compared to those stored at −20 and 4 °C. Although VC treatment was also stored at 24 °C, the color differences were not as significant as in MT and ST, because the greatest color difference for this treatment was immediately evidenced after processing. In apple products, the Δ*E* coefficient in purees treated by MT and ST and kept at −20 and 4 °C showed slight changes, which were lower than 3 up to the end of storage. However, HPP stored at 4 °C as well as ST kept at 24 °C exhibited the greatest color difference among all purees.

#### Sensory Changes during Storage

The influence of storage on the processed strawberry and apple samples on the sensory attributes (color, viscosity, aroma, flavor) as well as overall evaluation are shown in Tables S7 and S8, respectively. In strawberry products, storage temperature significantly affected the overall evaluation of the strawberry samples. During storage at −20 and 4 °C, all samples were considered acceptable, except for NT stored at −20 °C at the end of the storage period. Conversely, all of the samples stored at 24 °C fell below the level of acceptability after the second month of storage. In general, when comparing the MT and ST treatments during storage, higher scores were reported for ST.

In apple products, purees stored at −20 and 4 °C reached similar scores during storage. All of these samples were above the acceptability level (>5), except ST stored at 4 °C at the end of the shelf life. According to the overall evaluation, MT and ST stored at 24 °C were acceptable until the second month of storage, from the sixth month onward they scored below the acceptability level, reaching values very close to 0 at the end of storage. It was not possible to carry out the sensory evaluation protocol for the reprocessed samples due to health-related conditions derived from COVID-19. Therefore, these results are not reported here.

## Discussion

Most of the previous studies on the consequences of processing and storage on the nutritional value and bioactive compounds of food products has relied on laboratory experiments. The current research provides a more in-depth analysis of the extent to which storage conditions (temperature and time) impact the levels of bioactive phenolic compounds, color, and sensory attributes of strawberry and apple purees produced on an industrial scale using different processing techniques. Although the use of real industrial food production systems is expensive due to the large amount of fruit processed and time required to obtain the samples, it provides numerous advantages, such as being able to evaluate the real conditions and amounts with which the industry works, as well as improving nutrient retention^14^ resulting in nutritious products with an extended shelf life that allows them to be marketed along the value chain.^34^

In our study, the combined effect of the industrial food processing and storage of strawberry and apple purees was evaluated. Importantly, the exploratory factor analysis revealed that the storage temperature was the most influential factor in the degradation of phenolic compounds in the strawberry samples, the degradation of these compounds being more evident at higher storage temperatures, while in the apple samples, the processing techniques defined the grouping of the samples in two clusters, each one being influenced by the storage temperature. The main differences between both apple puree groups stem from the use of cold and hot crushing. In particular, the heat applied to the whole fruit before hot crushing (ST) industrial processing contributed to the release of flavonols and dihydrochalcones from the peel and seeds, whereas in MT and HPP industrial operations peel and seeds were removed after the cold crushing, which led to the loss of the compounds present in these structures.

### Influence of Storage on the Phenolic Composition of Strawberry Samples

In strawberry samples, proanthocyanidins were the most stable compounds, even under the more extreme conditions (24 °C, 12 months), whereas anthocyanins were highly degraded especially at 24 °C, and ellagitannins decreased under all conditions. In general, the different families of polyphenols were better preserved at −20 °C, and ST was the technique that led to less degradation. The explanation is probably that the higher thermal treatment applied inactivates the enzymes responsible for the polyphenol oxidation (PPO and POD). Initial losses of all polyphenols were previously observed just after processing when more severe thermal processing conditions were applied (vacuum concentration, VC).^10^

The high storage stability of proanthocyanidins has been evidenced in a laboratory scale experiment by Teleszko et al.^35^ A temperature dependent degradation was observed with losses (20–40%) of the flavan-3-ol monomers [(+)-catechin, (−)-epicatechin, and (−)-epigallocatechin gallate] in pasteurized strawberry cubes stored for 12 months at −20 °C, which were higher (40–70%) after storage for 2 months at 23 °C.^36^ These losses were more relevant than those found in our experiments at −20 and 24 °C, probably because only monomers were quantified in this previous study, while several reports have shown that catechin and epicatechin are very good substrates for polyphenol oxidase.^37,38^ Teleszko et al.^35^ also treated cloudy strawberry juice thermally. Contrary to our results, when the juice was kept at 4 °C, increases from 5 to 30% were observed. This increase could be explained by the protective effect exerted by the food matrix since cloudy juice contains pectin, which formed colloidal suspensions that limited their degradation. In general, Oszmiański and co-workers^39^ reported a better preservation of (+)-catechin and proanthocyanidins in clear, cloudy, and puree strawberry juices stored at 4 °C than in those kept at 30 °C. However, the concentration of proanthocyanidins during storage was less degraded in the purees followed by cloudy and clear juices in this order. Interestingly, the behavior of catechin and proanthocyanidins was also cultivar-dependent. Overall, these results evidenced that not only the storage conditions but also the cultivar and the food matrix influence the preservation of proanthocyanidins.

Ellagitannins were highly degraded, even in samples stored at −20 °C. Similar losses of ellagitannins were observed in MT and ST samples at the end of storage for all of the temperatures along with an increase in ellagic acid under all conditions. This can be explained by their hydrolysis^35,39^ or the binding of ellagitannins to cell wall polysaccharides or proteins.^40^ Previously, losses of ellagitannins were observed in blackberry juice stored at 5 °C for 35 days (with an increase in the degradation) when the storage temperature was increased.^41^ The behavior of ellagitannins during storage has been found to be highly dependent on the food matrix.^40,42^ Unlike our results, Hager et al.^40^ reported no differences on ellagitannin levels during 6 months of storage in IQF blackberries at −20 °C and blackberry clear juice and puree at 25 °C, but a degradation up to 42.5% was observed in cloudy blackberry juice. Similarly, Aaby et al.^42^ did not find differences in ellagitannin content in strawberry puree after 4 months of storage at 6 and 22 °C. Nonetheless, significant losses of 14 and 27% were recorded in the purees enriched with achenes stored at 6 and 22 °C, respectively. Consistent with our results, the concentration of ellagic acid increased progressively during storage at both temperatures. While several studies have reported increases of ellagic acid during storage, mainly attributed to ellagitannin depolymerization into ellagic acid,^39,43^ others found a degradation of ellagic acid attributed to nonenzymatic oxidative reactions in strawberries stored for 3 months at 30 °C^36^ and 12 months at −20 °C.^44^

Importantly, anthocyanin degradation was highly affected by storage temperature (particularly at 24 °C). The best storage temperature to preserve anthocyanins was −20 °C, and the best treatment was IQF. Although the percentage of loss of anthocyanins was lower in ST samples, the initial amount was slightly lower due to the stronger thermal treatment than in the MT, and the total amount observed after storage was similar for MT and ST purees. This is consistent with previous studies which have reported the instability of anthocyanins during storage in a variety of food matrices,^23,35,36,44^ as a result of oxidative reactions, triggered by insufficient inactivation of PPO, or partial reactivation of PPO during storage. Another factor influencing anthocyanin stability is the self-association mechanism, which suggests a positive effect on anthocyanin stability when increasing the concentration or in food matrixes with high anthocyanin content.^23^ Consistent with our study, extant research has reported losses of anthocyanins higher than 90% when different thermally treated strawberry products were stored above 20 °C for at least 3 months,^24,35,36,45,46^ while less degradation of anthocyanins was observed at lower storage temperatures and was dependent on the fruit variety.^23,24,35,46^

### Influence of Storage on the Phenolic Composition of Apple Samples

In apple puree, phenolic compounds were generally most stable during storage compared to in strawberry purees, and the highest degradations were observed in samples stored at 24 °C, which might be attributed to oxidative and nonoxidative reactions caused by the high storage temperature. Similar to strawberry samples, ST better preserved the phenolic compounds at all temperatures, due to the application of a more intense thermal treatment that could have better inactivated the PPO and POD enzymes and limited the degradation during storage.^47^ Similarly, the reprocessed apple puree samples, especially the RP.MT, preserved the compounds better at 24 °C than their nonreprocessed samples. However, the final concentration was in most cases similar or even lower because they generally started from lower concentrations of polyphenols with respect to their nonreprocessed samples. A high degradation was also observed for all polyphenols in HPP purees stored at 4 °C. This might be related to oxidation reactions derived from the PPO and POD remaining activities due to insufficient inactivation during pressurization.^48^

In general, similar to prior research,^49,50^ hydroxycinnamic acids were well preserved during frozen (−20 °C) and refrigerated storage (4 °C). At 24 °C, a higher degradation was observed in MT compared to ST, which increased over time. This can be attributed to the action of PPO, more present in MT samples, as chlorogenic acid is a good substrate for this enzyme.^49^ In line with our results, van der Sluis et al.^49^ found no significant differences in chlorogenic acid levels in apple juice stored at 4 and 20 °C for 1 month. However, after 7 months of storage at 20 °C, they observed a decline of about 40%. Likewise, in chokeberry juice stored at room temperature (25 °C), Wilkes et al.^51^ reported no differences in total hydroxycinnamic acids (chlorogenic and neochlorogenic acids) up to 3 months. However, progressive degradations were found after 4 months, ending up with 34% degradation in the sixth month. In our study, only small losses (5%) of hydroxycinnamic acids were observed in HPP samples stored at 4 °C. These results contrast with those obtained on a pilot plant scale, which found a high hydroxycinnamic acid degradation (higher 50%), mainly chlorogenic acid, in HPP apple juice (300 to 600 MPa/5 min) after 12 weeks of refrigerated storage,^52^ mostly explained by the lack of thermal inactivation of oxidative enzymes during HPP processing.

Proanthocyanidins were well preserved during the first 2 months of storage at any temperature, and even a slight increase was observed in ST samples. Previous studies have shown stable levels of catechins in pasteurized apple samples,^47,49,51^ whereas increases of 42% and 13% on catechin concentrations were reported in heat-treated apple juice after 2 weeks of storage at room temperature.^47^ After 6 months, the content of proanthocyanidins decreased, especially in the samples kept at 24 °C, in line with prior research.^19^ Similarly, degradation on catechin, epicatechin, procyanidin B1, procyanidin C1, and polymeric proanthocyanidins were reported in pasteurized apple purees after 6 months of storage at 30 °C.^20^ In addition, a higher degree of polymerization was observed in all of the purees as the storage progressed.^20^ In our study, consistent with prior research,^48,52^ we found a high degradation (24.4%) in industrially processed HPP samples stored at 4 °C. This might be caused by the lack of thermal treatment for enzyme inactivation.

Dihydrochalcones were the most stable compounds under all storage conditions with losses below 25%. Again, industrial ST showed the lowest storage degradation at all temperatures, and this degradation increased with temperature. Phloridzin was particularly stable to oxidation reactions, its degradation being mainly due to nonoxidative pathways.^49^ Similarly, Maragò et al.^47^ did not find significant differences in phloretin and phloridzin levels after 2 weeks of storage at room temperature in apple juice. In other studies, no significant differences were observed in phloridzin concentration in pasteurized apple juice kept at 4 and 20 °C for 1 month and even after 6 months in the absence of oxygen.^49^ Additionally, Oszmianski and Wojdyło^53^ found that phoretin-2′-O-xylosylglucoside and phloretin-2′-O-glucoside were stable after 6 months of storage at 30 °C in thermally treated apple purees, although a decrease of 18% in phloretin-2′-O-glucoside was reported in the puree from a different variety. The highest degradation was observed in HPP samples stored at 4 °C (23.7%). A more severe degradation of phloridzin (around 71–84%) was reported in HPP apple juice stored at 4 °C for 12 weeks,^48,52^ and this was probably due to the lack of thermal inactivation of enzymes (PPO).

Flavonols followed similar trends to dihydrochalcones, with no major changes during the storage of ST purees at −20 and 4 °C and only slight losses in the storage at 24 °C. This could be explained by the presence of quercetin glycosides, which have been reported as the less heat labile phenolic compounds in apples.^49,54^ In agreement with our results, a previous study^20^ reported no significant differences or minimal changes in the quercetin-3-O-rutinoside, quercetin-3-O-galactoside, quercetin-3-O-glucoside, quercetin-3-O-xyloside, quercetin-3-O-arabinoside, and quercetin-3-O-rhamnoside contents, in thermally treated apple puree after 6 months of storage at 30 °C. Another study recorded no significant differences in quercetin-3-galactoside in apple juice kept at room temperature after 2 weeks of storage, but it was conditioned by the variety.^47^ Similarly, van der Sluis et al.^49^ found no changes in total quercetin glycosides in apple juice kept at 4 and 20 °C for 1 month. Conversely, in our study, a higher degradation of flavonols was observed in MT samples, increasing with the temperature, and the higher losses were observed in HPP samples (69%).

### Color and Sensory Changes in Strawberry and Apple Samples during Storage

Storage also affected the color and sensory evaluation of the different samples. In strawberries, the change in color difference in MT and ST could be due to an anthocyanin discoloration caused by the condensation reactions of anthocyanins with ascorbic acid.^45^ On the other hand, in the sample that had no heat treatment applied, these changes might be due to some enzymatic oxidation.^24^ In the case of ST, it is important to take into account that after the heat treatment, anthocyanins are converted into colorless carbinol base and chalcone forms, therefore the strawberry puree became paler. Δ*E* values were lower than 5 up to 6 months of storage in the purees stored at −20 and 4 °C. However, the storage at 24 °C led to substantial increments on Δ*E* values throughout the storage period. The color changes in the samples stored at 24 °C are particularly attributed to Maillard nonenzymatic reactions induced by the storage conditions.^46^

As for the impact of storage on the sensory attributes, overall evaluation was considered as an indicator of general acceptability. Samples stored at −20 and 4 °C were scored over the acceptable limit established (5), except NT at −20 °C at the end of storage, which became watery. This change in firmness might be due to the effect of pectin methyl esterase activity on the tissue.^55^ However, samples kept at 24 °C were under the acceptability limit from the second month of storage, which was expected due to the changes observed in color reported above.

In apples, the higher color change observed in MT and HPP stored at 4 and 24 °C could be attributed to enzymatic browning as a result of residual oxidative enzymes (PPO and POD), which have also been related to the development of off-flavor compounds.^20,48,56^ Complete inactivation of PPO, POD, and PME has been extensively described after standard treatment.^57^ The most significant increment in Δ*E* was recorded for ST (6.4) puree kept at 24 °C, which could be partially attributed to nonenzymatic oxidation triggered by the high storage temperature.^58^ The overall sensory evaluation in the apple purees stored at −20 and 4 °C was similar and over the acceptability limit through the storage period. HPP kept at 4 °C fell slightly under the acceptability limit at the end of storage, which could be the result of some darkening in the color and loss of viscosity due oxidative and pectin methyl esterase enzymes.^48^ Interestingly, MT and ST purees kept at 24 °C scored over the acceptability limit up to the second month of storage but got the lowest score at the end of storage, which was expected due to the large changes in color.

This study provides new insights into the detrimental consequences of some storage conditions in comparison to processing with special emphasis on strawberry purees. In this case, the storage conditions had a stronger impact on the degradation of polyphenols and quality characteristics as compared to the conventional processing techniques used on an industrial scale. Proanthocyanidins were the main phenolics group and the most stable, whereas anthocyanins, being heat labile compounds, were the most affected as a result of processing and storage conditions. Samples stored at −20 and 4 °C ranked over the acceptability limit in the overall sensory evaluation, while samples stored at 24 °C showed higher degradation both in phenolic levels and in quality attributes. On the contrary, in the case of apple purees, industrial processing techniques had a stronger impact on their phenolic levels as compared to storage conditions. In this context, the standard industrial thermal treatment is recommended, as it obtained the best results in terms of the preservation of the polyphenol concentration after both processing and storage. However, the mild thermal treatment and HPP resulted in similar outcomes regarding the preservation of polyphenols during storage.

Overall, our results indicate that the stability of polyphenols in strawberries and apples was different in terms of processing and storage. That is to say, not at all fruits responded equally to different techniques and conditions. Interestingly, a lower degree of processing did not always lead to higher preservation of polyphenols during storage, and in some cases processing (as evidenced in apples) had a positive impact on some phenolics groups that are also stable during storage. Furthermore, although both processing and storage conditions are interrelated, as evidenced in this study, storage conditions have a stronger influence than processing in preserving the natural content of polyphenols in strawberries. Accordingly, manufacturers need to acknowledge the distinct behavior of each fruit to ensure the maintenance of polyphenol content in sensitive fruits such as strawberries and, at the same time, to select the ideal storage conditions (time and temperature) to minimize losses during their shelf life. Thus, there is an opportunity to adapt processing and storage conditions based on the fruit characteristics. The selection of the optimal processing techniques and adequate storage conditions would not only help consumers to take in meaningful quantities of bioactive compounds from processed fruits but also might stimulate further research to shed light on the current narratives and controversy around processing of foods.

## Supporting Information Available

(Table S1) phenolic compounds quantified in strawberry and apple samples; (Table S2) strawberry purees’ principal phenolic compounds after processing; (Table S3) strawberry purees’ minor phenolic compounds after processing; (Table S4) apple purees’ phenolic compounds after processing; (Table S5) color parameters (L*, Chroma, °Hue) and total color difference (Δ*E*) in strawberry purees after processing; (Table S6) color parameters (L*, Chroma, °Hue) and total color difference (Δ*E*) in apple purees after processing; (Table S7) sensory attributes (aroma, viscosity, flavor, color, overall evaluation assessed in a 9-point hedonic scale) in strawberry purees after processing; (Table S8) sensory attributes (aroma, viscosity, flavor, color assessed in a 9-point hedonic scale) in apple purees after processing (PDF)
