# Supplementary material for: Effect of Storage Conditions on the Stability of Polyphenols of Apple and Strawberry Purees Produced at Industrial Scale by Different Processing Techniques
Source: J Agric Food Chem. 2023 Jan 27;71(5):2541–53. doi: 10.1021/acs.jafc.2c07828 (PMC9912326; doi:10.1021/acs.jafc.2c07828)
Supplement: Supplementary file 1 — jf2c07828_si_001.pdf [file jf2c07828_si_001.pdf]

**Supplementary materials**

# Effect of storage conditions on the stability of polyphenols of apple and strawberry purees produced at industrial scale by different processing techniques

Gabriela L. Salazar-Orbea [a]; Rocío García-Villalba [a]; María J. Bernal [b,c]; Alberto Hernández [d]; Jose A. Egea [a]; Francisco A. Tomás-Barberán [a,*]; Luis M. Sánchez-Siles [b,c]

[a] Quality, Safety and Bioactivity of Plant-Derived Foods, Centro de Edafología y Biología Aplicada del Segura-Consejo Superior de Investigaciones Científicas (CEBAS-CSIC), 30100 Murcia [d], 30100, Spain; glsalazar@cebas.csic.es (G.L.S.-O.); rgvillalba@cebas.csic.es (R.G.-V); jaegea@cebas.csic.es (J.A.E)

[b] Research and Nutrition Department, Hero Group, 30820 Alcantarilla, Spain; luisma.sanchez@hero.es (L.M.S-S.); mjose.bernal@hero.es (M.J.B)

[c] Institute for Research and Nutrition, Hero Group, 5600 Lenzburg, Switzerland

[d] AMC Natural Drinks Group, 30100 Murcia, Spain; alhernandez@amunoz.com (A.H)

* Corresponding author: Phone: +34-968396200 (ext. 6334); E-mail address: fatomas@cebas.csic.es (F.A. Tomás-Barberán)

**Supplementary materials (Tables S1-S8)**

**Table S1.** Phenolic compounds quantified in strawberry and apple samples.

| Phenolic group | Strawberry compounds | Apple compounds |
|---|---|---|
| **Anthocyanins** | Cyanidin-3-glucoside<br>Pelargonidin-3-glucoside<br>Pelargonidin-3-rutinoside | |
| **Ellagitannins** | Bis-HHDP-glucose<br>HHDP-galloyl-glucose<br>Galloyl-bis-HHDP-glucose | |
| **Ellagic acid and conjugates** | Ellagic acid deoxyhexoside<br>Ellagic acid | |
| **Dihydrochalcones** | | Phloretin-2-0-ylosylglucoside<br>Phloridzin |
| **Hydroxycinnamic acids** | p-Coumaric acid hexoside<br>Ferulic acid hexose derivative | Chlorogenic acid<br>4-0-Caffeoylquinic acid<br>1-Caffeoylquinic acid<br>4-p-Coumaroylquinic acid |
| **Flavonols** | Quercetin-3-glucuronide | Quercetin-3-0-rutinoside<br>Quercetin-3-0-glucoside<br>Quercetin-3-0-galactoside<br>Quercetin-3-0-xyloside<br>Quercetin-3-0-arabinoside<br>Quercetin-3-0-rhamnoside |
| **Flavan 3-ols**<br>**(after phloroglucinolysis)** | (+)-Catechin<br>(-)-Epicatechin<br>Afzelechin | (+)-Catechin<br>(-)-Epicatechin |

HHDP: Hexahydroxydiphenic acid

**Table S2.** Strawberry purees principal phenolic compounds after processing by individual quick freezing (IQF), NT: no heat treatment (NT), mild treatment (MT), standard treatment (ST), vacuum concentration (VC) and after storage for 2, 6 and 12 months at -20, 4, and 24 °C. Different letters within each storage temperature indicate significant differences (p<0.05) according to one-way ANOVA followed by Tukey's comparison test. % DG.

| Storage T° | Treatment | Storage | Ellagitannins | | % DG Storage | Anthocyanins | | % DG Storage | Proanthocyanidins | | mDP | % DG Storage | Total polyphenols | | % DG Storage |
|---|---|---|---|---|---|---|---|---|---|---|---|---|---|---|---|
| -20°C | IQF | After processing | 50.52 ± 1.27 | bc | | 19.66 ± 0.60 | bc | | 97.97 ± 1.81 | e | 3.8 | | 178.00 ± 0.81 | cd | |
| | | 2 months | 33.83 ± 2.80 | fg | ↓33.03% | 21.45 ± 0.13 | a | ↑9.10% | 97.97 ± 1.81 | e | 3.8 | ≈% | 163.36 ± 1.55 | f | ↓8.22% |
| | | 6 months | 20.81 ± 0.77 | i | ↓58.80% | 19.91 ± 0.20 | bc | ↑1.27% | 90.64 ± 0.36 | f | 4.2 | ↓7.48% | 140.86 ± 0.97 | h | ↓20.86% |
| | | 12 months | 19.47 ± 0.53 | i | ↓61.46% | 18.57 ± 0.10 | d | ↓5.54% | 87.33 ± 1.37 | f | 4.2 | ↓10.86% | 135.80 ± 1.56 | h | ↓23.70% |
| | NT | After processing | 47.35 ± 0.68 | cd | | 20.52 ± 0.33 | b | | 112.52 ± 5.50 | ab | 4.5 | | 189.25 ± 5.20 | ab | |
| | | 2 months | 30.40 ± 0.73 | gh | ↓35.79% | 16.35 ± 0.14 | ef | ↓20.32% | 112.52 ± 5.50 | ab | 4.2 | ≈% | 168.45 ± 5.28 | ef | ↓10.99% |
| | | 6 months | 17.67 ± 0.34 | i | ↓62.68% | 15.66 ± 0.11 | fg | ↓23.68% | 105.83 ± 1.53 | bcd | 4.1 | ↓5.94% | 149.03 ± 1.42 | g | ↓21.25% |
| | | 12 months | 17.28 ± 0.87 | i | ↓63.50% | 13.02 ± 0.31 | i | ↓36.54% | 99.41 ± 1.47 | de | 4.2 | ↓11.65% | 137.88 ± 2.86 | h | ↓27.14% |
| | MT | After processing | 53.42 ± 1.32 | ab | | 19.38 ± 0.41 | c | | 111.86 ± 1.64 | ab | 3.9 | | 194.93 ± 2.72 | a | |
| | | 2 months | 44.24 ± 1.50 | d | ↓17.18% | 18.41 ± 0.14 | d | ↓5.00% | 111.32 ± 2.31 | ab | 3.8 | ↓0.48% | 184.88 ± 2.03 | bc | ↓5.15% |
| | | 6 months | 35.90 ± 1.25 | ef | ↓32.79% | 16.79 ± 0.35 | e | ↓13.36% | 102.24 ± 1.73 | cde | 3.8 | ↓8.60% | 166.39 ± 1.68 | ef | ↓14.64% |
| | | 12 months | 31.82 ± 0.63 | gh | ↓40.43% | 14.59 ± 0.35 | h | ↓24.71% | 105.76 ± 2.57 | bcd | 3.6 | ↓5.45% | 162.58 ± 3.73 | f | ↓16.59% |
| | ST | After processing | 54.47 ± 1.11 | a | | 15.29 ± 0.21 | gh | | 113.07 ± 1.24 | a | 3.5 | | 193.11 ± 1.15 | a | |
| | | 2 months | 47.95 ± 0.72 | c | ↓11.96% | 16.64 ± 0.24 | e | ↑8.82% | 113.42 ± 1.75 | a | 3.2 | ↑0.30% | 188.52 ± 2.81 | ab | ↓2.37% |
| | | 6 months | 37.45 ± 1.27 | e | ↓31.24% | 16.04 ± 0.11 | efg | ↑4.90% | 107.82 ± 1.52 | abc | 3.3 | ↓4.64% | 172.89 ± 0.49 | de | ↓10.47% |
| | | 12 months | 29.81 ± 0.92 | h | ↓45.27% | 13.70 ± 0.28 | i | ↓10.39% | 113.28 ± 0.77 | a | 3.3 | ↑0.18% | 167.14 ± 1.01 | ef | ↓13.44% |
| 4°C | MT | After processing | 53.42 ± 1.32 | ab | | 19.38 ± 0.41 | a | | 111.86 ± 1.64 | b | 3.9 | | 194.93 ± 2.72 | a | |
| | | 2 months | 50.32 ± 3.55 | b | ↓5.80% | 15.42 ± 0.34 | b | ↓20.43% | 112.39 ± 0.75 | b | 3.4 | ↑0.47% | 189.44 ± 3.55 | a | ↓2.81% |
| | | 6 months | 34.32 ± 0.29 | d | ↓35.75% | 10.27 ± 0.24 | d | ↓47.00% | 90.36 ± 0.19 | d | 3.2 | ↓19.22% | 146.74 ± 0.52 | b | ↓24.72% |
| | | 12 months | 29.75 ± 0.88 | e | ↓44.30% | 5.87 ± 0.14 | f | ↓69.71% | 71.89 ± 1.09 | e | 3.2 | ↓35.73% | 117.96 ± 1.78 | c | ↓39.48% |
| | ST | After processing | 54.47 ± 1.11 | a | | 15.29 ± 0.21 | b | | 113.07 ± 1.24 | b | 3.5 | | 193.11 ± 1.15 | a | |
| | | 2 months | 56.66 ± 0.37 | a | ↑4.02% | 13.00 ± 0.28 | c | ↓14.97% | 112.71 ± 0.62 | b | 3.2 | ↓0.31% | 195.31 ± 0.84 | a | ↑1.13% |
| | | 6 months | 46.21 ± 1.55 | c | ↓15.16% | 9.68 ± 0.04 | d | ↓36.69% | 122.98 ± 2.20 | a | 3.0 | ↑8.76% | 192.36 ± 3.71 | a | ↓0.38% |
| | | 12 months | 28.79 ± 0.77 | e | ↓47.14% | 6.99 ± 0.16 | e | ↓54.28% | 105.38 ± 1.88 | c | 2.9 | ↓6.80% | 151.70 ± 2.65 | b | ↓21.44% |
| 24°C | MT | After processing | 53.42 ± 1.32 | a | | 19.38 ± 0.41 | a | | 111.86 ± 1.64 | a | 3.9 | | 194.93 ± 2.72 | a | |
| | | 2 months | 32.83 ± 1.58 | cd | ↓38.54% | 2.84 ± 0.03 | c | ↓85.34% | 112.39 ± 0.75 | a | 3.3 | ↑0.47% | 159.65 ± 1.99 | c | ↓18.09% |
| | | 6 months | 23.87 ± 0.40 | e | ↓55.31% | 0.80 ± 0.02 | e | ↓95.87% | 73.59 ± 0.90 | d | 3.3 | ↓34.21% | 108.20 ± 0.59 | f | ↓44.49% |
| | | 12 months | 21.13 ± 0.37 | ef | ↓60.44% | 0.00 ± 0.00 | f | ↓100% | 69.76 ± 0.40 | e | 3.1 | ↓37.63% | 100.68 ± 0.31 | g | ↓48.35% |
| | ST | After processing | 54.47 ± 1.11 | a | | 15.29 ± 0.21 | b | | 113.07 ± 1.24 | a | 3.5 | | 193.11 ± 1.15 | a | |
| | | 2 months | 42.87 ± 2.25 | b | ↓21.29% | 3.24 ± 0.26 | c | ↓78.8% | 112.71 ± 0.62 | a | 3.1 | ↓0.31% | 170.99 ± 3.19 | b | ↓11.45% |
| | | 6 months | 31.72 ± 0.82 | cd | ↓41.76% | 1.20 ± 0.01 | de | ↓92.15% | 106.71 ± 2.50 | b | 3.0 | ↓5.62% | 151.35 ± 3.29 | d | ↓21.62% |
| | | 12 months | 21.53 ± 0.06 | e | ↓60.47% | 1.71 ± 0.00 | d | ↓88.81% | 94.56 ± 0.59 | c | 2.9 | ↓16.37% | 126.04 ± 0.59 | e | ↓34.73% |
| | VC | After processing | 34.26 ± 0.46 | c | | 2.83 ± 0.18 | c | | 45.09 ± 1.46 | f | 2.5 | | 88.51 ± 1.69 | h | |
| | | 2 months | 30.04 ± 1.29 | d | ↓12.31% | 0.00 ± 0.00 | f | ↓100% | 45.09 ± 1.46 | f | 2.3 | ↓1.42% | 82.76 ± 0.27 | i | ↓6.49% |
| | | 6 months | 18.23 ± 0.27 | fg | ↓46.78% | 0.00 ± 0.00 | f | ↓100% | 32.43 ± 0.49 | g | 2.3 | ↓28.07% | 57.41 ± 0.74 | j | ↓35.13% |
| | | 12 months | 17.45 ± 0.12 | g | ↓49.06% | 0.00 ± 0.00 | f | ↓100% | 34.92 ± 0.27 | g | 2.1 | ↓22.55% | 59.70 ± 0.36 | j | ↓32.54% |

Different letters within each storage temperature indicate significant differences (p<0.05) according to one-way ANOVA followed by Tukey's comparison test. Being "a" the sample with the higher value within each group. % DG, Degradation percentage.

**Table S3.** Strawberry purees minor phenolic compounds after processing by individual quick freezing (IQF), NT: no heat treatment (NT), mild treatment (MT), standard treatment (ST), vacuum concentration (VC) and after storage for 2, 6 and 12 months at -20, 4, and 24°C.

| Storage T° | Treatment | Storage | Ellagic acid & derivatives | | % DG Storage | Hydroxycinnamic acids | | % DG Storage | Flavonols | | % DG Storage |
|---|---|---|---|---|---|---|---|---|---|---|---|
| -20°C | IQF | After processing | 0.91 ± 0.02 | g | | 6.89 ± 0.08 | ab | | 2.01 ± 0.06 | def | |
| | | 2 months | 1.48 ± 0.04 | ef | ↑62.63% | 6.88 ± 0.04 | ab | ↓0.14% | 1.71 ± 0.02 | efg | ↓14.92% |
| | | 6 months | 1.40 ± 0.12 | ef | ↑53.84% | 6.44 ± 0.11 | c | ↓6.53% | 1.64 ± 0.22 | g | ↓18.40% |
| | | 12 months | 1.30 ± 0.03 | f | ↑42.85% | 6.28 ± 0.05 | cd | ↓8.85% | 2.83 ± 0.02 | a | ↑40.79% |
| | NT | After processing | 1.52 ± 0.05 | ef | | 5.29 ± 0.01 | h | | 2.03 ± 0.04 | de | |
| | | 2 months | 4.18 ± 0.03 | a | ↑175% | 3.62 ± 0.04 | j | ↓31.56% | 1.37 ± 0.02 | g | ↓32.51% |
| | | 6 months | 3.54 ± 0.02 | b | ↑132.89% | 4.66 ± 0.09 | i | ↓11.90% | 1.65 ± 0.06 | fg | ↓18.71% |
| | | 12 months | 3.55 ± 0.23 | b | ↑133.55% | 3.09 ± 0.06 | k | ↓41.58% | 1.50 ± 0.01 | g | ↓26.10% |
| | MT | After processing | 1.48 ± 0.10 | ef | | 6.46 ± 0.09 | c | | 2.31 ± 0.16 | cd | |
| | | 2 months | 2.02 ± 0.32 | c | ↑36.48% | 6.79 ± 0.04 | b | ↑5.10% | 2.07 ± 0.26 | cde | ↓10.38% |
| | | 6 months | 2.05 ± 0.06 | c | ↑38.51% | 7.08 ± 0.08 | a | ↑9.59% | 2.31 ± 0.07 | cd | ≈ |
| | | 12 months | 2.15 ± 0.02 | c | ↑45.27% | 5.87 ± 0.07 | ef | ↓9.13% | 2.35 ± 0.09 | cd | ↑1.73% |
| | ST | After processing | 1.68 ± 0.12 | de | | 5.74 ± 0.08 | fg | | 2.84 ± 0.17 | a | |
| | | 2 months | 1.98 ± 0.05 | cd | ↑17.85% | 6.07 ± 0.07 | de | ↑5.74% | 2.44 ± 0.05 | bc | ↓14.08% |
| | | 6 months | 2.08 ± 0.05 | c | ↑23.80% | 6.70 ± 0.04 | b | ↑16.72% | 2.78 ± 0.05 | ab | ↓2.11% |
| | | 12 months | 1.99 ± 0.07 | cd | ↑18.45% | 5.55 ± 0.01 | g | ↓3.31% | 2.78 ± 0.07 | ab | ↓2.11% |
| 4°C | MT | After processing | 1.48 ± 0.10 | d | | 6.46 ± 0.09 | c | | 2.31 ± 0.16 | c | |
| | | 2 months | 2.25 ± 0.06 | b | ↑52.02% | 7.04 ± 0.01 | ab | ↑8.97% | 1.99 ± 0.05 | d | ↓13.85% |
| | | 6 months | 2.46 ± 0.01 | b | ↑66.21% | 7.20 ± 0.05 | a | ↑11.45% | 2.09 ± 0.03 | cd | ↓9.52% |
| | | 12 months | 2.46 ± 0.09 | b | ↑66.21% | 5.90 ± 0.09 | d | ↓8.66% | 2.08 ± 0.05 | cd | ↓9.95% |
| | ST | After processing | 1.68 ± 0.12 | c | % | 5.74 ± 0.08 | d | % | 2.84 ± 0.17 | b | % |
| | | 2 months | 2.78 ± 0.04 | a | ↑65.47% | 6.90 ± 0.18 | b | ↑20.20% | 3.23 ± 0.15 | a | ↑13.73% |
| | | 6 months | 2.99 ± 0.03 | a | ↑77.97% | 6.95 ± 0.04 | ab | ↑21.08% | 3.53 ± 0.02 | a | ↑24.29% |
| | | 12 months | 2.44 ± 0.08 | b | ↑45.23% | 5.31 ± 0.02 | e | ↓7.49% | 2.78 ± 0.07 | b | ↓2.11% |
| 24°C | MT | After processing | 1.48 ± 0.10 | h | | 6.46 0.09 | a | | 2.31 0.16 | cd | |
| | | 2 months | 3.40 ± 0.06 | a | ↑129.72% | 6.24 0.05 | b | ↓3.40% | 1.93 0.08 | e | ↓16.45% |
| | | 6 months | 2.51 ± 0.02 | d | ↑69.59% | 5.46 0.10 | d | ↓15.47% | 1.95 0.02 | e | ↓15.58% |
| | | 12 months | 3.21 ± 0.02 | ab | ↑116.89% | 4.53 0.06 | e | -29.87% | 2.04 0.04 | de | ↓11.68% |
| | ST | After processing | 1.68 ± 0.12 | g | | 5.74 0.08 | c | | 2.84 0.17 | b | |
| | | 2 months | 2.80 ± 0.08 | c | ↑66.66% | 6.24 0.06 | b | ↑8.71% | 3.10 0.14 | ab | ↑9.15% |
| | | 6 months | 2.72 ± 0.06 | cd | ↑61.90% | 5.69 0.03 | c | ↓0.87% | 3.29 0.01 | a | ↑15.84% |
| | | 12 months | 1.91 ± 0.04 | ef | ↑13.69% | 3.92 0.01 | f | ↓31.70% | 2.38 0.02 | c | ↓16.19% |
| | VC | After processing | 0.73 ± 0.02 | i | | 4.05 0.05 | f | | 1.53 0.03 | f | |
| | | 2 months | 2.14 ± 0.02 | e | ↑193.15% | 3.07 0.10 | g | ↓24.19% | 2.40 0.06 | c | ↑56.86% |
| | | 6 months | 1.72 ± 0.13 | fg | ↑135.61% | 2.78 0.04 | h | ↓31.35% | 2.23 0.07 | cde | ↑45.75% |
| | | 12 months | 3.06 ± 0.07 | b | ↑319.17% | 2.01 0.02 | i | ↓50.37% | 2.24 0.04 | cde | ↑46.40% |

Different letters within each storage temperature indicate significant differences ($p<0.05$) according to one-way ANOVA followed by Tukey's comparison test. Being "a" the sample with the higher value within each group. % DG, Degradation percentage.

**Table S4.** Apple purees phenolic compounds after processing by mild treatment (MT), high pressure processing (HPP), standard treatment (ST), mild treatment + re-processing (RP.MT), standard treatment + re-processing (RP.ST) and after storage for 2, 6 and 12 months at -20, 4, and 24°C.

| Storage T° | Treatment | Storage | Dihydro-chalcones | | % DG Storage | Hydroxyci-nnamic acids | | % DG Storage | Flavonols | | % DG Storage | Proanthocyanidins | | % DG Storage | Total Polyphenols | | % DG Storage |
|---|---|---|---|---|---|---|---|---|---|---|---|---|---|---|---|---|---|
| -20°C | MT | After processing | 13.46 ± 0.30 | d | | 51.36 ± 1.28 | a | | 4.25 ± 0.06 | d | | 38.39 ± 1.31 | ab | | 107.4 ± 1.31 | c | |
| | | 2 months | 12.99 ± 0.01 | d | ↓3.49% | 49.16 ± 0.08 | bc | ↓4.28% | 4.01 ± 0.01 | d | ↓5.64% | 39.94 ± 0.86 | a | ↑4.03% | 106.1 ± 0.76 | c | ↓1.21% |
| | | 6 months | 11.82 ± 0.08 | e | ↓12.18% | 48.06 ± 0.22 | c | ↓6.42% | 3.13 ± 0.01 | e | ↓26.35% | 32.66 ± 0.47 | d | ↓14.92% | 95.68 ± 0.51 | d | ↓10.91% |
| | | 12 months | 11.42 ± 0.11 | e | ↓15.15% | 48.00 ± 0.23 | c | ↓6.54% | 1.93 ± 0.02 | f | ↓54.58% | 33.33 ± 0.96 | d | ↓13.18% | 94.70 ± 0.69 | d | ↓11.82% |
| | ST | After processing | 22.56 ± 0.15 | b | | 45.55 ± 0.91 | d | | 17.82 ± 0.23 | c | | 36.75 ± 1.07 | bc | | 122.6 ± 0.17 | b | |
| | | 2 months | 23.98 ± 0.21 | a | ↑6.29% | 49.98 ± 0.58 | ab | ↑9.72% | 19.26 ± 0.14 | a | ↑8.08% | 40.57 ± 0.82 | a | ↑10.39% | 133.8 ± 1.38 | a | ↑9.13% |
| | | 6 months | 21.80 ± 0.09 | c | ↓3.36% | 47.42 ± 0.40 | c | ↑4.1% | 17.51 ± 0.23 | c | ↓1.73% | 34.05 ± 0.35 | d | ↓7.34% | 120.8 ± 0.98 | b | ↓1.46% |
| | | 12 months | 21.88 ± 0.22 | c | ↓3.01% | 47.89 ± 0.36 | c | ↑5.13% | 18.27 ± 0.26 | b | ↑2.52% | 34.44 ± 0.22 | cd | ↓6.28% | 122.4 ± 0.99 | b | ↓0.16% |
| 4°C | HPP | After processing | 13.14 ± 0.27 | c | | 46.96 ± 0.31 | bcd | | 3.51 ± 0.09 | d | | 37.25 ± 1.31 | ab | | 100.8 ± 1.83 | d | |
| | | 2 months | 12.02 ± 0.08 | d | ↓8.52% | 45.40 ± 0.26 | cd | ↓3.32% | 3.28 ± 0.02 | d | ↓6.55% | 35.06 ± 0.51 | bc | ↓5.87% | 95.78 ± 0.78 | ef | ↓4.98% |
| | | 6 months | 12.22 ± 0.30 | d | ↓7.00% | 47.25 ± 1.14 | bc | ↑0.61% | 3.09 ± 0.03 | d | ↓11.96% | 29.66 ± 0.18 | ef | ↓20.37% | 92.25 ± 1.62 | f | ↓8.48% |
| | | 12 months | 10.02 ± 0.03 | e | ↓23.74% | 44.52 ± 0.15 | d | ↓5.19% | 1.08 ± 0.01 | f | ↓69.23% | 28.16 ± 0.43 | f | ↓24.40% | 83.80 ± 0.26 | g | ↓16.86% |
| | MT | After processing | 13.46 ± 0.30 | d | | 51.36 ± 1.28 | a | | 4.25 ± 0.06 | d | | 38.39 ± 1.31 | ab | | 107.4 ± 1.31 | c | |
| | | 2 months | 12.29 ± 0.02 | d | ↓8.69% | 46.39 ± 0.09 | bcd | ↓9.67% | 3.43 ± 0.02 | d | ↓19.29% | 36.55 ± 0.67 | ab | ↓4.79% | 98.66 ± 0.78 | de | ↓8.13% |
| | | 6 months | 11.97 ± 0.38 | d | ↓11.06% | 48.37 ± 1.47 | b | ↓5.82% | 3.16 ± 0.04 | d | ↓25.64% | 32.72 ± 0.54 | cd | ↓14.76% | 96.25 ± 1.96 | ef | ↓10.38% |
| | | 12 months | 11.63 ± 0.14 | d | ↓13.59% | 46.25 ± 0.35 | bcd | ↓9.94% | 2.18 ± 0.01 | e | ↓48.70% | 34.89 ± 0.54 | bc | ↓9.11% | 94.96 ± 0.05 | ef | ↓11.58% |
| | ST | After processing | 22.56 ± 0.15 | b | | 45.55 ± 0.91 | d | | 17.82 ± 0.23 | c | | 36.75 ± 1.07 | bc | | 122.6 ± 0.17 | b | |
| | | 2 months | 22.33 ± 0.09 | a | ↓1.01% | 45.94 ± 0.41 | bcd | ↑0.85% | 17.84 ± 0.03 | a | ↑0.11% | 38.26 ± 0.56 | a | ↑4.1% | 124.3 ± 0.31 | a | ↑1.38% |
| | | 6 months | 21.82 ± 0.73 | ab | ↓3.28% | 47.17 ± 1.67 | bc | ↑3.55% | 17.20 ± 0.65 | b | ↓3.47% | 31.55 ± 0.49 | de | ↓14.14% | 117.7 ± 2.98 | b | ↓3.99% |
| | | 12 months | 21.11 ± 0.05 | b | ↓6.42% | 46.14 ± 0.23 | bcd | ↑1.29% | 17.59 ± 0.07 | ab | ↓1.29% | 33.01 ± 1.98 | cd | ↓10.17% | 117.8 ± 1.93 | b | ↓3.91% |
| 24°C | MT | After processing | 13.46 ± 0.30 | d | | 51.36 ± 1.28 | a | | 4.25 ± 0.06 | d | | 38.39 ± 1.31 | ab | | 107.4 ± 1.31 | c | |
| | | 2 months | 12.26 ± 0.159 | g | ↓8.91% | 45.84 ± 0.563 | cd | ↓10.74% | 3.38 ± 0.04 | h | ↓20.47% | 35.40 ± 0.458 | ab | ↓7.788% | 96.9 ± 0.87 | e | ↓9.77% |
| | | 6 months | 11.87 ± 0.268 | gh | ↓11.81% | 44.84 ± 1053 | de | ↓12.69% | 3.04 ± 0.01 | hi | ↓28.47% | 29.86 ± 0.214 | cd | ↓22.21% | 89.6 ± 1.46 | gh | ↓16.57% |
| | | 12 months | 10.83 ± 0.136 | j | ↓19.53% | 35.99 ± 0.649 | j | ↓29.92% | 1.12 ± 0.04 | k | ↓73.64% | 22.68 ± 0.186 | f | ↓40.92% | 70.6 ± 0.76 | k | ↓34.26% |
| | ST | After processing | 22.56 ± 0.15 | b | | 45.55 ± 0.91 | d | | 17.82 ± 0.23 | c | | 36.75 ± 1.07 | bc | | 122.6 ± 0.17 | b | |
| | | 2 months | 22.66 ± 0.237 | a | ↑0.44% | 47.35 ± 0.242 | bc | ↑3.95% | 17.96 ± 0.17 | a | ↑0.78% | 38.26 ± 0.909 | a | 4.1% | 126.2 ± 1.49 | a | ↑2.93% |
| | | 6 months | 20.80 ± 0.240 | c | ↓7.80% | 42.02 ± 0.484 | fg | ↓7.74% | 16.05 ± 0.19 | b | ↓9.93% | 29.63 ± 0.615 | cd | ↓19.37% | 108.5 ± 1.45 | c | ↓11.50% |
| | | 12 months | 19.78 ± 0.304 | d | ↓12.32% | 36.62 ± 0.312 | ij | ↓19.60% | 14.95 ± 0.24 | c | ↓16.10% | 24.27 ± 2989 | ef | ↓33.95% | 95.6 ± 2.33 | ef | ↓22.02% |
| 24°C | RP.MT | After processing | 11.57 ± 0.112 | hi | | 47.82 ± 0.824 | b | | 2.97 ± 0.02 | i | | 30.60 ± 0.746 | c | | 92.9 ± 1.45 | efg | |
| | | 2 months | 11.07 ± 0.048 | ij | ↓4.32% | 44.45 ± 0.464 | de | ↓7.04% | 3.24 ± 0.14 | hi | ↑9.09% | 29.45 ± 0.566 | cd | ↓3.75% | 88.2 ± 0.12 | h | ↓5.05% |
| | | 6 months | 10.69 ± 0.086 | jk | ↓7.60% | 42.94 ± 0.292 | ef | ↓10.20% | 2.12 ± 0.00 | j | ↓28.61% | 26.40 ± 0.313 | def | ↓13.72% | 82.1 ± 0.54 | i | ↓11.62% |
| | | 12 months | 10.14 ± 0.210 | k | ↓12.35% | 40.32 ± 0.723 | gh | ↓15.68% | 2.16 ± 0.01 | j | ↓27.27% | 23.38 ± 1791 | f | ↓23.59% | 76.0 ± 2.40 | j | ↓18.19% |
| 24°C | RP.ST | After processing | 21.38 ± 0.045 | b | | 46.28 ± 0.132 | bcd | | 15.14 ± 0.07 | c | | 32.15 ± 0.726 | bc | | 114.9 ± 0.74 | b | |
| | | 2 months | 20.28 ± 0.039 | cd | ↓5.144% | 42.95 ± 0.245 | ef | ↓7.19% | 13.73 ± 0.04 | d | ↓9.31% | 29.25 ± 1665 | cd | ↓9.02% | 106.2 ± 1.49 | cd | ↓7.57% |
| | | 6 months | 20.14 ± 0.122 | d | ↓5.79% | 41.61 ± 0.203 | fg | ↓10.09% | 12.94 ± 0.06 | e | ↓14.53% | 28.23 ± 0.137 | cde | ↓12.19% | 102.9 ± 0.28 | d | ↓10.44% |
| | | 12 months | 15.33 ± 0.111 | e | ↓28.29% | 38.52 ± 0.374 | hi | ↓16.76% | 11.86 ± 0.10 | f | ↓21.66% | 25.90 ± 3069 | def | ↓19.44% | 91.6 ± 2.52 | fgh | ↓20.27% |

Different letters within each storage temperature indicate significant differences ($p<0.05$) according to one-way ANOVA followed by Tukey's comparison test. % DG. Being “a” the sample with the higher value within each group. Degradation percentage.

**Table S5.** Color parameters (L*, Chroma, °Hue) and total color difference (ΔE) in strawberry purees after processing by Individual Quick Freezing (IQF), NT: no heat treatment (NT), mild treatment (MT), standard treatment (ST), vacuum concentration (VC) and after storage for 2, 6 and 12 months at -20, 4 and 24°C.

| Storage temp | Treatment | Storage | L* | | Chroma | | °Hue | | ΔE |
|---|---|---|---|---|---|---|---|---|---|
| -20°C | IQF | After processing | 33.77 + 0.02 | b | 31.27 + 0.21 | c | 32.65 + 0.46 | c | |
| | | 2 months | 33.78 + 0.01 | b | 31.28 + 0.15 | c | 32.90 + 0.18 | c | 0.18 + 0.09 |
| | | 6 months | 34.56 + 0.01 | a | 32.94 + 0.06 | b | 33.80 + 0.06 | b | 1.95 + 0.05 |
| | | 12 months | 34.91 + 0.55 | a | 36.31 + 0.62 | a | 34.81 + 0.26 | a | 5.34 + 0.49 |
| | NT | After processing | 25.10 + 0.22 | c | 25.73 + 0.05 | a | 31.22 + 0.07 | b | |
| | | 2 months | 26.78 + 0.02 | a | 25.05 + 0.10 | b | 30.56 + 0.14 | b | 1.83 + 0.04 |
| | | 6 months | 25.57 + 0.01 | b | 23.05 + 0.11 | c | 33.50 + 0.37 | a | 2.89 + 0.05 |
| | | 12 months | 23.79 + 0.12 | d | 20.07 + 0.12 | d | 34.84 + 1.31 | a | 5.99 + 0.21 |
| | MT | After processing | 34.43 + 1.06 | b | 28.99 + 1.10 | a | 33.20 + 0.38 | d | |
| | | 2 months | 37.63 + 0.01 | a | 27.88 + 0.09 | a | 34.06 + 0.04 | c | 3.41 + 0.01 |
| | | 6 months | 36.50 + 0.04 | a | 27.96 + 0.26 | a | 35.59 + 0.29 | b | 2.60 + 0.19 |
| | | 12 months | 36.57 + 0.00 | a | 28.05 + 0.04 | a | 36.79 + 0.30 | a | 2.94 + 0.09 |
| | ST | After processing | 35.62 + 1.06 | a | 26.97 + 0.39 | b | 31.01 + 0.51 | c | |
| | | 2 months | 33.85 + 0.05 | bc | 30.18 + 0.02 | a | 34.44 + 0.07 | b | 4.04 + 0.01 |
| | | 6 months | 32.71 + 0.02 | c | 30.73 + 0.07 | a | 36.62 + 0.25 | a | 5.53 + 0.02 |
| | | 12 months | 34.49 + 0.02 | ab | 30.61 + 0.04 | a | 37.18 + 0.08 | a | 4.91 + 0.01 |
| 4°C | MT | After processing | 34.43 + 1.06 | b | 28.99 + 1.10 | a | 33.20 + 0.38 | d | |
| | | 2 months | 36.00 + 0.00 | a | 29.65 + 0.02 | a | 34.77 + 0.11 | c | 1.88 + 0.01 |
| | | 6 months | 35.85 + 0.02 | ab | 26.28 + 0.06 | b | 37.59 + 0.66 | b | 3.73 + 0.21 |
| | | 12 months | 35.70 + 0.05 | ab | 25.02 + 0.11 | b | 48.87 + 0.54 | a | 8.44 + 0.26 |
| | ST | After processing | 35.82 + 1.06 | b | 27.05 + 0.37 | a | 30.92 + 0.51 | c | |
| | | 2 months | 34.64 + 0.00 | b | 27.38 + 0.17 | a | 33.87 + 0.19 | b | 1.75 + 0.12 |
| | | 6 months | 35.85 + 0.01 | b | 26.01 + 0.01 | b | 39.31 + 0.23 | a | 4.01 + 0.10 |
| | | 12 months | 39.26 + 0.01 | a | 25.87 + 0.08 | b | 38.53 + 0.04 | a | 5.18 + 0.02 |
| 24°C | MT | After processing | 34.43 + 1.06 | b | 28.99 + 1.10 | a | 33.20 + 0.38 | d | |
| | | 2 months | 38.60 + 0.08 | a | 24.67 + 0.00 | b | 57.44 + 0.04 | c | 12.73 + 0.01 |
| | | 6 months | 37.26 + 0.01 | a | 23.31 + 0.21 | b | 62.28 + 0.09 | b | 14.52 + 0.00 |
| | | 12 months | 37.53 + 0.03 | a | 23.21 + 0.04 | b | 65.87 + 0.49 | a | 16.00 + 0.20 |
| | ST | After processing | 35.62 + 1.06 | c | 26.97 + 0.39 | a | 31.01 + 0.51 | d | |
| | | 2 months | 39.71 + 0.03 | b | 23.65 + 0.13 | bc | 44.32 + 0.45 | c | 7.92 + 0.1 |
| | | 6 months | 40.34 + 0.06 | b | 24.19 + 0.07 | b | 50.42 + 0.57 | a | 10.25 + 0.2 |
| | | 12 months | 42.66 + 0.06 | a | 23.27 + 0.15 | c | 49.07 + 0.22 | b | 11.22 + 0.0 |
| | VC | After processing | 32.88 + 0.01 | c | 20.73 + 0.12 | a | 52.14 + 0.25 | a | |
| | | 2 months | 32.89 + 0.03 | c | 20.84 + 0.10 | a | 51.68 + 0.40 | a | 0.22 + 0.1 |
| | | 6 months | 33.76 + 0.00 | b | 16.31 + 0.01 | c | 49.91 + 0.13 | b | 4.56 + 0.0 |
| | | 12 months | 36.58 + 0.05 | a | 17.38 + 0.07 | b | 50.52 + 0.27 | b | 5.03 + 0.0 |

At each storage temperature, different letters within each treatment indicate significant differences ($p<0.05$) according to one-way ANOVA followed by Tukey's comparison test. Being “a” the sample with the higher value within each group.

**Table S6**. Color parameters (L*, Chroma, °Hue) and total color difference (ΔE) in apple purees after processing by mild treatment (MT), high pressure processing (HPP), standard treatment (ST), mild treatment + re-processing (RP.MT), standard treatment + re-processing (RP.ST) and after storage for 2, 6 and 12 months at -20, 4 and 24°C.

| Storage temp | Treatment | Storage | L* | | Chroma | | ºHue | | ΔE |
|---|---|---|---|---|---|---|---|---|---|
| -20°C | MT | After processing | 48.92 ± 0.35 | b | 16.61 ± 0.14 | b | 114.75 ± 0.05 | a | 0.00 ± 0.00 |
| | | 2 months | 49.52 ± 0.00 | a | 17.07 ± 0.12 | a | 113.65 ± 0.07 | b | 0.82 ± 0.05 |
| | | 6 months | 49.56 ± 0.13 | a | 16.82 ± 0.07 | ab | 112.66 ± 0.60 | c | 0.91 ± 0.17 |
| | | 12 months | 47.16 ± 0.07 | c | 16.85 ± 0.03 | ab | 113.89 ± 0.09 | b | 1.79 ± 0.06 |
| | ST | After processing | 50.08 ± 0.26 | b | 18.33 ± 0.16 | c | 109.66 ± 0.08 | b | 0.00 ± 0.00 |
| | | 2 months | 50.21 ± 0.03 | b | 19.10 ± 0.22 | a | 111.10 ± 0.57 | a | 0.93 ± 0.07 |
| | | 6 months | 51.53 ± 0.02 | a | 18.79 ± 0.03 | ab | 108.58 ± 0.04 | c | 1.55 ± 0.00 |
| | | 12 months | 48.26 ± 0.03 | c | 18.70 ± 0.03 | bc | 109.22 ± 0.04 | bc | 1.85 ± 0.03 |
| 4°C | MT | After processing | 48.92 ± 0.35 | b | 16.61 ± 0.14 | a | 114.75 ± 0.05 | b | 0.00 ± 0.00 |
| | | 2 months | 50.46 ± 0.27 | a | 15.09 ± 0.09 | b | 114.81 ± 0.01 | b | 2.18 ± 0.12 |
| | | 6 months | 47.26 ± 0.14 | c | 13.03 ± 0.07 | c | 115.17 ± 0.06 | a | 3.95 ± 0.12 |
| | | 12 months | 49.41 ± 0.11 | b | 14.90 ± 0.06 | b | 112.14 ± 0.02 | c | 1.92 ± 0.02 |
| | HPP | After processing | 48.57 ± 0.28 | b | 16.75 ± 0.18 | a | 114.49 ± 0.10 | a | 0.00 ± 0.00 |
| | | 2 months | 48.76 ± 0.21 | b | 13.44 ± 0.12 | c | 111.62 ± 0.07 | b | 3.40 ± 0.10 |
| | | 6 months | 49.83 ± 0.43 | a | 14.45 ± 0.20 | b | 111.74 ± 0.05 | b | 2.75 ± 0.17 |
| | | 12 months | 45.19 ± 0.17 | c | 10.13 ± 0.10 | d | 114.45 ± 0.02 | a | 7.42 ± 0.04 |
| | ST | After processing | 50.08 ± 0.26 | a | 18.33 ± 0.16 | a | 109.66 ± 0.08 | ab | 0.00 ± 0.00 |
| | | 2 months | 50.13 ± 0.16 | a | 17.82 ± 0.59 | a | 111.94 ± 2.36 | a | 1.18 ± 0.19 |
| | | 6 months | 48.01 ± 0.12 | c | 16.98 ± 0.10 | b | 108.56 ± 0.04 | b | 2.49 ± 0.16 |
| | | 12 months | 48.82 ± 0.09 | b | 15.65 ± 0.01 | c | 108.99 ± 0.02 | ab | 2.97 ± 0.04 |
| 24°C | MT | After processing | 48.92 ± 0.35 | a | 16.61 ± 0.14 | a | 114.75 ± 0.05 | a | 0.00 ± 0.00 |
| | | 2 months | 47.48 ± 0.35 | b | 12.15 ± 0.08 | c | 114.40 ± 0.08 | b | 4.69 ± 0.18 |
| | | 6 months | 47.66 ± 0.08 | b | 13.25 ± 0.04 | b | 108.50 ± 0.03 | c | 3.94 ± 0.03 |
| | | 12 months | 44.63 ± 0.26 | c | 13.09 ± 0.04 | b | 102.61 ± 0.04 | d | 6.37 ± 0.18 |
| | ST | After processing | 50.08 ± 0.26 | a | 18.33 ± 0.16 | a | 109.66 ± 0.08 | a | 0.00 ± 0.00 |
| | | 2 months | 47.40 ± 0.09 | b | 16.94 ± 0.02 | b | 108.21 ± 0.05 | b | 3.04 ± 0.09 |
| | | 6 months | 47.71 ± 0.15 | b | 16.09 ± 0.06 | d | 107.62 ± 0.07 | c | 3.32 ± 0.14 |
| | | 12 months | 46.71 ± 0.18 | c | 16.70 ± 0.05 | c | 100.17 ± 0.03 | d | 4.73 ± 0.15 |
| | RP.MT | After processing | 46.36 ± 0.33 | c | 12.06 ± 0.05 | c | 114.41 ± 0.07 | a | |
| | | 2 months | 47.90 ± 0.10 | a | 12.86 ± 0.09 | b | 114.11 ± 0.04 | a | 1.74 ± 0.18 |
| | | 6 months | 46.76 ± 0.11 | bc | 12.60 ± 0.32 | b | 106.98 ± 2.16 | b | 1.99 ± 0.50 |
| | | 12 months | 47.11 ± 0.21 | b | 14.35 ± 0.13 | a | 105.99 ± 0.03 | b | 2.56 ± 0.01 |
| | RP.ST | After processing | 52.37 ± 0.16 | a | 16.47 ± 0.02 | c | 108.71 ± 0.05 | a | |
| | | 2 months | 52.10 ± 0.11 | ab | 16.39 ± 0.06 | c | 108.76 ± 0.12 | a | 0.28 ± 0.21 |
| | | 6 months | 51.91 ± 0.08 | b | 18.21 ± 0.85 | b | 104.15 ± 1.76 | b | 2.40 ± 0.44 |
| | | 12 months | 50.54 ± 0.09 | c | 19.37 ± 0.02 | a | 102.94 ± 0.01 | b | 3.81 ± 0.07 |

At each storage temperature, different letters within each treatment indicate significant differences ($p<0.05$) according to one-way ANOVA followed by Tukey's comparison test. Being “a” the sample with the higher value within each group.

**Table S7.** Sensory attributes (aroma, viscosity, flavor, color, overall evaluation assessed in a 9-point hedonic scale) in strawberry purees after processing (by Individual Quick Freezing (IQF), NT: no heat treatment (NT), mild treatment (MT), standard treatment (ST), vacuum concentration (VC) and after storage for 2, 6 and 12 months at -20°C, 4°C and 24°C.

| Storage T° | Treatment | Storage | Aroma | | Viscosity | | Flavor | | Color | | Overall evaluation |
|---|---|---|---|---|---|---|---|---|---|---|---|
| **-20°C** | IQF | After processing | 8.1 ± 0.7 | ab | 6.1 ± 0.9 | ab | 7.0 ± 1.2 | abc | 8.1 ± 0.8 | ab | 8.0 ± 0.8 |
| | | 2 months | 7.2 ± 1.6 | abcd | 6.8 ± 1.8 | ab | 6.6 ± 1.4 | abcd | 8.5 ± 0.7 | ab | 7.0 ± 1.3 |
| | | 6 months | 7.7 ± 1.1 | ab | 6.0 ± 1.1 | ab | 6.0 ± 0.7 | abcde | 8.1 ± 0.7 | ab | 6.6 ± 1.2 |
| | | 12 months | 5.8 ± 1.2 | def | 5.9 ± 1.1 | ab | 5.4 ± 1.6 | cdef | 8.1 ± 1.0 | abc | 6.2 ± 0.9 |
| | NT | After processing | 8.3 ± 0.9 | a | 5.4 ± 0.7 | bc | 7.2 ± 0.9 | ab | 8.6 ± 0.7 | a | 6.9 ± 1.5 |
| | | 2 months | 7.5 ± 1.4 | abc | 5.0 ± 0.7 | bc | 6.6 ± 1.2 | abcd | 7.9 ± 0.9 | abc | 5.9 ± 0.9 |
| | | 6 months | 7.7 ± 0.7 | ab | 1.8 ± 1.1 | d | 3.7 ± 1.7 | f | 6.8 ± 0.8 | cd | 5.7 ± 1.3 |
| | | 12 months | 5.1 ± 1.7 | f | 1.6 ± 1.5 | d | 4.5 ± 1.2 | ef | 7.7 ± 1.1 | abcd | 4.6 ± 0.9 |
| | MT | After processing | 8.2 ± 0.8 | a | 6.6 ± 1.2 | ab | 7.2 ± 1.1 | a | 8.4 ± 0.7 | ab | 7.5 ± 0.6 |
| | | 2 months | 7.1 ± 1.1 | abcde | 5.2 ± 1.1 | bc | 5.1 ± 0.9 | def | 6.6 ± 0.9 | d | 6.8 ± 0.9 |
| | | 6 months | 6.0 ± 0.7 | cdef | 3.8 ± 0.8 | c | 4.9 ± 0.8 | def | 7.3 ± 0.5 | abcd | 5.8 ± 1.2 |
| | | 12 months | 7.2 ± 1.0 | abcd | 5.9 ± 1.4 | ab | 7.2 ± 0.9 | ab | 7.2 ± 0.9 | bcd | 7.2 ± 0.8 |
| | ST | After processing | 7.0 ± 0.7 | abcde | 7.3 ± 1.1 | a | 6.8 ± 0.9 | abc | 6.8 ± 0.6 | d | 6.6 ± 1.0 |
| | | 2 months | 6.6 ± 1.2 | bcdef | 6.4 ± 0.9 | ab | 5.5 ± 1.5 | bcde | 6.4 ± 0.9 | d | 5.2 ± 1.3 |
| | | 6 months | 5.1 ± 1.2 | f | 6.3 ± 0.9 | ab | 5.7 ± 0.4 | bcde | 7.9 ± 0.9 | abc | 5.9 ± 0.6 |
| | | 12 months | 5.4 ± 0.7 | ef | 6.8 ± 1.0 | ab | 6.3 ± 0.7 | abcd | 7.9 ± 0.9 | abc | 6.3 ± 0.6 |
| 4°C | MT | After processing | 8.2 ± 0.8 | a | 6.6 ± 1.2 | abc | 7.2 ± 1.1 | a | 8.4 ± 0.7 | a | 7.5 ± 0.6 |
| | | 2 months | 7.7 ± 1.4 | ab | 5.5 ± 1.5 | bc | 6.0 ± 0.7 | ab | 7.7 ± 0.8 | ab | 7.0 ± 1.5 |
| | | 6 months | 5.5 ± 0.9 | cd | 5.2 ± 1.0 | c | 6.6 ± 0.9 | ab | 6.3 ± 0.8 | c | 5.1 ± 1.0 |
| | | 12 months | 5.4 ± 0.7 | d | 6.1 ± 1.1 | abc | 6.0 ± 1.6 | ab | 4.4 ± 1.2 | d | 5.9 ± 0.8 |
| | ST | After processing | 7.0 ± 0.7 | b | 7.3 ± 1.1 | a | 6.8 ± 0.9 | ab | 6.8 ± 0.6 | bc | 6.6 ± 1.0 |
| | | 2 months | 5.1 ± 1.4 | d | 5.2 ± 0.5 | c | 5.7 ± 1.3 | b | 6.6 ± 0.8 | bc | 6.5 ± 1.2 |
| | | 6 months | 6.7 ± 0.8 | bc | 6.7 ± 0.9 | abc | 6.2 ± 0.8 | ab | 6.6 ± 0.7 | bc | 6.4 ± 0.8 |
| | | 12 months | 5.8 ± 0.8 | cd | 7.0 ± 1.1 | ab | 5.7 ± 1.3 | b | 6.6 ± 1.1 | bc | 6.4 ± 0.5 |
| 24°C | MT | After processing | 8.2 ± 0.8 | a | 6.6 ± 1.2 | bc | 7.2 ± 1.1 | a | 8.4 ± 0.7 | a | 7.5 ± 0.6 |
| | | 2 months | 3.6 ± 0.8 | c | 3.4 ± 0.5 | d | 4.5 ± 1.9 | bc | 2.3 ± 1.4 | cd | 3.6 ± 1.2 |
| | | 6 months | 4.0 ± 1.1 | c | 3.0 ± 1.2 | d | 3.2 ± 0.7 | bcd | 1.8 ± 2.4 | cde | 2.5 ± 1.3 |
| | | 12 months | 3.0 ± 1.2 | c | 3.2 ± 1.0 | d | 1.9 ± 1.7 | d | 0.5 ± 0.8 | e | 2.2 ± 0.8 |
| | ST | After processing | 7.0 ± 0.7 | b | 7.3 ± 1.1 | ab | 6.8 ± 0.9 | a | 6.8 ± 0.6 | b | 6.6 ± 1.0 |
| | | 2 months | 3.7 ± 0.5 | c | 6.6 ± 0.8 | bc | 4.8 ± 0.8 | b | 2.1 ± 0.7 | cde | 2.3 ± 0.9 |
| | | 6 months | 3.9 ± 1.8 | c | 6.4 ± 1.4 | bc | 4.1 ± 0.6 | bc | 0.7 ± 0.9 | de | 3.1 ± 1.2 |
| | | 12 months | 4.0 ± 1.3 | c | 5.4 ± 1.0 | c | 2.9 ± 1.0 | cd | 2.7 ± 0.9 | c | 4.1 ± 0.6 |
| | VC | After processing | 6.0 ± 0.6 | b | 7.7 ± 0.8 | ab | 3.2 ± 1.4 | bcd | 1.6 ± 1.3 | cde | 4.5 ± 0.9 |
| | | 2 months | 3.2 ± 1.8 | c | 8.4 ± 0.8 | a | 3.4 ± 1.3 | bcd | 2.3 ± 0.8 | cd | 2.3 ± 1.2 |
| | | 6 months | 2.8 ± 1.9 | c | 7.2 ± 1.2 | ab | 4.1 ± 1.5 | bc | 1.6 ± 1.5 | cde | 3.6 ± 1.4 |
| | | 12 months | 2.7 ± 1.2 | c | 7.9 ± 0.8 | ab | 2.2 ± 1.0 | d | 0.5 ± 0.8 | e | 3.0 ± 1.3 |

At each storage temperature, different letters within each treatment indicate significant differences ($p<0.05$) according to one-way ANOVA followed by Tukey's comparison test. Being “a” the sample with the higher value within each group.

**Table S8.** Sensory attributes (aroma, viscosity, flavor, color assessed in a 9-point hedonic scale) in apple purees after processing by mild treatment (MT), high pressure processing (HPP), standard treatment (ST) and after storage for 2, 6 and 12 months at -20°C, 4°C and 24°C.

| Storage T° | Treatment | Storage | Aroma | | Viscosity | | Flavor | | Color | | Overall evaluation |
|---|---|---|---|---|---|---|---|---|---|---|---|
| -20°C | MT | After processing | 7.9 ± 0.9 | a | 7.0 ± 1.8 | a | 7.5 ± 1.8 | a | 7.8 ± 1.3 | a | 7.3 ± 1.8 |
| | | 2 months | 6.5 ± 1.5 | ab | 7.4 ± 1.3 | a | 7.1 ± 1.0 | a | 7.4 ± 0.8 | a | 7.4 ± 0.8 |
| | | 6 months | 4.6 ± 1.1 | ab | 6.3 ± 2.5 | a | 5.6 ± 3.0 | ab | 6.3 ± 2.5 | a | 5.7 ± 2.5 |
| | | 12 months | 8.0 ± 1.1 | a | 8.0 ± 1.1 | a | 8.0 ± 1.1 | a | 7.7 ± 0.9 | a | 8.0 ± 1.2 |
| | ST | After processing | 5.8 ± 1.7 | ab | 6.3 ± 1.8 | a | 6.3 ± 1.4 | ab | 7.2 ± 0.9 | a | 5.9 ± 1.9 |
| | | 2 months | 5.8 ± 1.2 | ab | 5.6 ± 1.9 | a | 5.4 ± 2.5 | ab | 6.4 ± 1.1 | a | 6.3 ± 1.5 |
| | | 6 months | 4.6 ± 1.5 | ab | 5.6 ± 2.3 | a | 6.0 ± 3.0 | ab | 6.0 ± 3.0 | a | 6.0 ± 3.0 |
| | | 12 months | 4.0 ± 2.8 | b | 4.2 ± 2.5 | a | 3.0 ± 1.8 | b | 5.7 ± 1.5 | a | 4.5 ± 0.6 |
| 4°C | HPP | After processing | 7.5 ± 1.5 | ab | 7.9 ± 1.1 | a | 8.2 ± 0.6 | a | 7.9 ± 0.8 | a | 7.9 ± 0.9 |
| | | 2 months | 6.7 ± 1.7 | ab | 7.3 ± 1.8 | a | 7.9 ± 1.2 | a | 7.1 ± 1.4 | a | 7.8 ± 1.3 |
| | | 6 months | 6.3 ± 2.0 | ab | 7.6 ± 1.1 | a | 6.3 ± 3.0 | ab | 7.0 ± 2.0 | ab | 4.0 ± 2.5 |
| | | 12 months | 4.5 ± 3.0 | b | 4.7 ± 3.3 | a | 4.5 ± 3.0 | b | 3.7 ± 2.9 | b | 4.8 ± 2.1 |
| | MT | After processing | 7.9 ± 0.9 | a | 7.0 ± 1.8 | a | 7.5 ± 1.8 | ab | 7.8 ± 1.3 | a | 7.3 ± 1.8 |
| | | 2 months | 6.5 ± 1.6 | ab | 7.0 ± 1.8 | a | 7.1 ± 1.5 | ab | 7.0 ± 0.9 | a | 7.5 ± 1.0 |
| | | 6 months | 5.3 ± 4.0 | ab | 7.3 ± 2.0 | a | 5.0 ± 4.3 | ab | 8.0 ± 1.0 | a | 4.0 ± 3.8 |
| | | 12 months | 8.5 ± 0.5 | a | 8.5 ± 0.5 | a | 8.5 ± 0.5 | a | 8.2 ± 0.5 | a | 8.5 ± 0.6 |
| | ST | After processing | 5.8 ± 1.7 | ab | 6.3 ± 1.8 | a | 6.3 ± 1.4 | ab | 7.2 ± 0.9 | a | 5.9 ± 1.9 |
| | | 2 months | 6.8 ± 0.7 | ab | 6.4 ± 1.5 | a | 7.4 ± 0.8 | ab | 7.1 ± 0.7 | a | 7.1 ± 1.1 |
| | | 6 months | 7.3 ± 0.5 | ab | 7.3 ± 1.5 | a | 6.0 ± 1.7 | ab | 7.6 ± 1.1 | a | 4.0 ± 1.2 |
| | | 12 months | 6.7 ± 0.9 | ab | 7.2 ± 1.7 | a | 7.0 ± 1.4 | ab | 7.5 ± 1.9 | a | 7.3 ± 1.3 |
| 24°C | MT | After processing | 7.9 ± 0.9 | a | 7.0 ± 1.8 | a | 7.5 ± 1.8 | a | 7.8 ± 1.3 | a | 7.3 ± 1.8 |
| | | 2 months | 6.5 ± 1.1 | ab | 6.9 ± 1.2 | a | 6.3 ± 1.4 | a | 6.3 ± 1.7 | a | 6.7 ± 1.2 |
| | | 6 months | 2.0 ± 1.7 | c | 5.0 ± 3.4 | ab | 2.3 ± 2.3 | b | 1.0 ± 0.0 | b | 2.0 ± 1.7 |
| | | 12 months | 1.7 ± 1.5 | c | 2.2 ± 2.5 | b | 1.2 ± 0.5 | b | 1.2± 0.5 | b | 1.0 ± 1.0 |
| | ST | After processing | 5.8 ± 1.7 | b | 6.3 ± 1.8 | a | 6.3 ± 1.4 | a | 7.2 ± 0.9 | a | 5.9 ± 1.9 |
| | | 2 months | 6.6 ± 1.5 | ab | 6.6 ± 2.4 | a | 6.7 ± 2.0 | a | 7.3 ± 1.0 | a | 7.0 ± 1.7 |
| | | 6 months | 4.0 ± 1.7 | bc | 5.6 ± 2.3 | ab | 4.0 ± 2.6 | ab | 2.6 ± 1.5 | b | 4.3 ± 1.2 |
| | | 12 months | 1.2 ± 0.5 | c | 1.0 ± 0.0 | b | 1.2 ± 0.5 | b | 1.0 ± 0.0 | b | 1.3 ± 0.5 |

At each storage temperature, different letters within each treatment indicate significant differences ($p<0.05$) according to one-way ANOVA followed by Tukey's comparison test. Being “a” the sample with the higher value within each group.
